# Structure and interaction of therapeutic proteins in solution: a combined simulation and experimental study

**DOI:** 10.1080/00268976.2023.2236248

**Published:** 2023-07-17

**Authors:** Suman Saurabh, Zongyi Li, Peter Hollowell, Thomas Waigh, Peixun Li, John Webster, John M. Seddon, Cavan Kalonia, Jian R. Lu, Fernando Bresme

**Affiliations:** a Department of Chemistry, Molecular Sciences Research Hub Imperial College, London, United Kingdom; b Biological Physics Group, School of Physics and Astronomy, Faculty of Science and Engineering, The University of Manchester, Manchester, UK; c Dosage Form Design and Development, BioPharmaceutical Development, BioPharmaceuticals R&D, AstraZeneca, Gaithersburg, MD, USA; d Photon Science Institute, The University of Manchester, Manchester, UK; e STFC ISIS Facility, Rutherford Appleton Laboratory, Didcot, UK

**Keywords:** Monoclonal antibodies, virial coefficient, potential of mean force, molecular dynamics simulations

## Abstract

The aggregation of therapeutic proteins in solution has attracted significant interest, driving efforts to understand the relationship between microscopic structural changes and protein-protein interactions determining aggregation processes in solution. Additionally, there is substantial interest in being able to predict aggregation based on protein structure as part of molecular developability assessments. Molecular Dynamics provides theoretical tools to complement experimental studies and to interrogate and identify the microscopic mechanisms determining aggregation. Here we perform all-atom MD simulations to study the structure and inter-protein interaction of the Fab and Fc fragments of the monoclonal antibody (mAb) COE3. We unravel the role of ion-protein interactions in building the ionic double layer and determining effective inter-protein interaction. Further, we demonstrate, using various state-of-the-art force fields (charmm, gromos, amber, opls/aa), that the protein solvation, ionic structure and protein-protein interaction depend significantly on the force field parameters. We perform SANS and Static Light Scattering experiments to assess the accuracy of the different forcefields. Comparison of the simulated and experimental results reveal significant differences in the forcefields' performance, particularly in their ability to predict the protein size in solution and inter-protein interactions quantified through the second virial coefficients. In addition, the performance of the forcefields is correlated with the protein hydration structure.

## GRAPHICAL ABSTRACT

**Figure:**
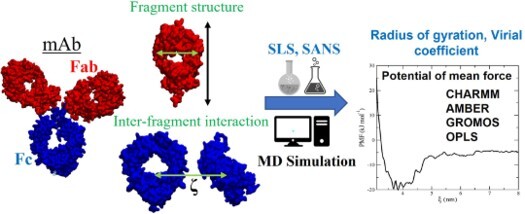

## 1. Introduction

Natural and recombinant proteins have recently gained popularity as therapeutic agents in drug discovery, with many commercialised protein products available in the market [1]. One of the major issues faced during the manufacture and storage of therapeutic proteins is their aggregation.

One of the most important variables determining the solution stability is the intrinsic aggregation propensity of the protein, which quantifies the tendency of proteins to form aggregates. Protein aggregation reduces antibody product quality, which could impact efficacy. Moreover, the presence of aggregates in some circumstances has been linked to increased immunogenicity, making it essential to monitor and control aggregation [2]. High-concentration protein formulations are often required to support dosing via subcutaneous injection. Aggregation rates are usually increased at high concentrations, which can pose a challenge during product development [3].

Proteins are believed to aggregate through short-range interactions between hydrophobic and uncharged or charged regions of partially unfolded proteins. Quantifying the interaction between folded proteins as a function of distance can provide microscopic insight into the aggregation mechanism and help identify residues and regions which could potentially be modified to reduce aggregation. Specifically, protein aggregation takes place when aggregation-prone regions on the protein surface interact with each other. In solution, proteins undergo intermittent unfolding at the secondary and tertiary structure level, resulting in either transient or irreversible exposure of the aggregation hot spots at the protein surface. Undistorted proteins also form small reversible oligomeric clusters, which may precede subsequent unfolding of the protein, followed by aggregation [4–6].

The kinetics of protein aggregation depend strongly on the composition of the protein formulation. Addition of  salt or change in the solution pH can modify the rate of aggregation [7]. Increasing salt concentration leads to the screening of protein charge, reducing double layer repulsion [7]. Change in the pH leads to modification of the protein charge and concomitant changes in double-layer interactions. Furthermore, changes in the pH modify the intra-protein electrostatic interactions, potentially influencing the tendency of aggregation-prone regions to get exposed to the solvent and buffer [7]. The molecular-level understanding of protein aggregation is still limited, and current approaches to reduce aggregation rates rely on generating experimental data, which is time-consuming. Hence, understanding protein-specific mechanisms involved in protein aggregation at the molecular level could help accelerate product development.

Molecular dynamics (MD) simulations have gained popularity as a tool to explain the molecular mechanism behind various biological processes. The increase in computational power has expanded the applicability of MD simulations to the study of large proteins [8–10] in atomistic detail, enabling MD to support experimental studies in the field of drug discovery and delivery [11–16]. The determination of the aggregation tendency of therapeutic proteins using MD simulations relies on using accurate force fields (*ff* s) that can predict protein interactions, structure and dynamics as a function of the solution composition. Here we report all-atom MD simulations of the Fab and Fc fragments of the monoclonal antibody (mAb) COE3 [17]. We investigate the protein structure, solvation structure, ionic environment around the protein, and inter-protein interactions, using several state-of-the-art *ff* s (amber, charmm, gromos, opls/aa ). Our work highlights essential differences in the predictions obtained with different *ff* s, and provides insights into the microscopic mechanisms influencing the aggregation of these complex proteins.

## 2. Materials and methods

### 2.1. Protein models and simulation conditions

We simulated the Fab and Fc fragments of mAb COE3. The sequence of the Fc fragment of COE3 is identical to the Fc fragment of the human IGG B12 (pdb id: 1HZH). The sequence of COE3 Fab fragment differs by 27 % from the IGG. Hence, the initial structure of the Fc fragment was obtained by removing the two Fab fragments of the 1HZH structure. The initial structure of the Fab fragment was obtained from Ref. [17]. mAb COE3, like any other antibody, consists of two light chains (LCs) and two heavy chains (HCs). The two LCs are 215 residue-long proteins, while the HCs are 450 residues long. Each Fab fragment consists of the complete light chain (LC ${}_{1-215}$) and a part of the heavy chain (HC ${}_{1-229}$). The Fab structure has 5 disulfide bonds, 1 interchain and 4 intrachain (2 each in the HC and LC parts). The Fc fragment consists of HC ${}_{229-450}$ of the two heavy chains and has 6 disulfide bonds in its structure (4 intra-chain and 2 inter-chain). The parts of the LC and HC constituting the Fab and Fc fragments, the structure of the Fab and Fc fragments and the position of the disulfide bonds are shown in Figure S1 of Supplementary Information (SI).

The simulations were performed at conditions corresponding to pH=7. propKa3.1 [18,19] was used to determine the protonation states of the proteins. We obtained a net charge of +11e (resulting from a neutral GLU residue) for the Fab fragment and +2e (resulting from three doubly-protonated HIS residues), for the Fc fragment. The position of the amino acids that were protonated are shown in Figure S1 of SI. After choosing the appropriate protonation states for the different titrable amino acid residues, the proteins were solvated with water in a cubic box of side 12 nm. The net charge of the Fab and Fc systems was neutralised using 11 and 2 Cl ${}^{-}$ ions, respectively. An additional 148 Na ${}^{+}$ and Cl ${}^{-}$ ions were added to achieve a salt concentration of 150 mM. The final Fab and Fc systems contained 54225 and 54099 water molecules, respectively.

The *ff* parameters used to describe the various inter-, and intra-molecular interactions in the simulations are listed in Table 1. The default ion parameters (Dang-Smith for the Cl ${}^{-}$ ion [20] in combination with the Åqvist [21] parameters for the Na ${}^{+}$ ions) were used with the amber99sb-ildn *ff* [23]. The gromos96-54a7 *ff* [24] is a modified version of the gromos96-53a6 [25] ff. The latter has been optimised, targeting the solvation-free energy of amino acid analogues. The gromos96-54a7 contains some corrections to the torsional terms to improve the treatment of *α*-helical regions [25]. For this *ff* , we considered two water models (*wm*s): the spc [26,27] model, which has been used to parametrise the gromos *ff* , and the spce model [26], which provides a better description of the thermodynamics, structure and dynamics of liquid water. The simulations with the charmm *ff* [28] were performed with the recommended tips3p *wm*, and with tip3p [26,27] water. The latter model uses point charges for the hydrogens with no Lennard-Jones (*lj*) interaction, whereas the tips3p model was parametrised by adding *lj* interactions with the hydrogens in the water molecule. These two models, tip3p and tips3p, have similar surface tension, dielectric constant and self-diffusion coefficient [29]. With the opls/aa *ff* [30], we used the tip4p *wm* [27] (used to parameterise the original forcefield). In addition, to understand the impact of the *wm* on the protein solvation, we also used the spce *wm*. Table 1 summarises all the *ff* parameter combinations considered in this work, and the ion parameters are listed in Table 2.

**Table 1.** *ff* /wm/ion parameters used in this work.

Protein	*ff*	*wm*	ion parameter
Fab	amber99sb-ildn	tip3p	Dang-Smith (Cl ${}^{-}$) [20] + Åqvist (Na ${}^{+}$) [21]
	charmm27	tip3p	charmm22
	charmm27	tips3p	charmm22
	gromos96 54a7	spce	Reif-Hunenberger [22]
	gromos96 54a7	spc	Reif-Hunenberger
	opls/aa	spce	opls
	opls/aa	tip4p	opls
Fc	amber99sb-ildn	tip3p	Dang-Smith (Cl ${}^{-}$) + Åqvist (Na ${}^{+}$)
	charmm27	tip3p	charmm22
	charmm27	tips3p	charmm22
	gromos96 54a7	spce	Reif-Hunenberger
	gromos96 54a7	spc	Reif-Hunenberger
	opls/aa	spce	opls
	opls/aa	tip4p	opls

**Table 2.** Details of the ion-parameters used in this work. *σ* is the van der Waals radius and *ϵ* refers to the well depth of the *lj* interaction.

	amber (default)		charmm22		R-H		OPLS	
Ion	*σ*	*ϵ*	*σ*	*ϵ*	*σ*	*ϵ*	*σ*	*ϵ*
Na ${}^{+}$	0.3328	0.01159	0.24299	0.19623	0.31213	0.02215	0.33305	0.01160
Cl ${}^{-}$	0.4401	0.41840	0.40447	0.6276	0.40957	0.67844	0.44172	0.49283

### 2.2. Simulation protocol

All the simulations were performed using the GROMACS(2021.3) package [31,32]. The initial configurations were minimised using the steepest descent method with all the protein atoms held fixed with harmonic restraints (force constant, $1000\;\mathrm{kJ}/(\mathrm{mol}\;{\mathrm{nm}}^{2}$)) to their initial positions to remove bad contacts with the water molecules and ions. Following the minimization, the systems were pre-equilibrated for 1 ns in the NVT ensemble at 300 K, keeping the protein atoms restrained at their initial positions. After pre-equilibration, the systems were subjected to a 1 ns long unrestrained equilibration in the NPT ensemble at 300 K, and 1 bar. Following the equilibration, we performed 200 ns long production runs in the NPT ensemble. For each of the seven *ff*-*wm* combinations, we performed three independent runs (for both Fab and Fc) resulting in a cumulative simulation time of 8.4 μs. All the simulations were performed with the canonical v-rescale thermostat [33] (coupling constant 0.5 ps). For the equilibration phase, we used the Berendsen barostat [34], with a pressure coupling constant of 0.5 ps, and for the production phase we employed the Parrinello-Rahman barostat [35] (coupling constant of 2.0 ps). The electrostatic interactions were computed using the Particle Mesh Ewald [36] method. We employed a cut-off of 1 nm for the dispersion interactions, and dispersion corrections were included in calculating the pressure. An integration time step of 2 fs was employed in all the simulations, and the bonds involving hydrogens were held rigid using the LINCS algorithm [37].

### 2.3. Quantification of the protein size

The size of the proteins was quantified using the radius of gyration (R ${}_{g}$),

(1) $$R_{g}^{2}=\frac{1}{M}\Sigma_{i=1}^{i=N}m_{i}(\vec{r_{i}}-\vec{R}{)}^{2}$$

where *N* is the total number of atoms in the protein, *M* is the total mass of the protein, $\Sigma_{i}^{N}m_{i}$, where $m_{i}$ is the mass of the *i*th atom, and $\vec{r_{i}}$ is atomic position. $\vec{R}$ defines the position of the center of mass of the protein.

**Figure 1.**
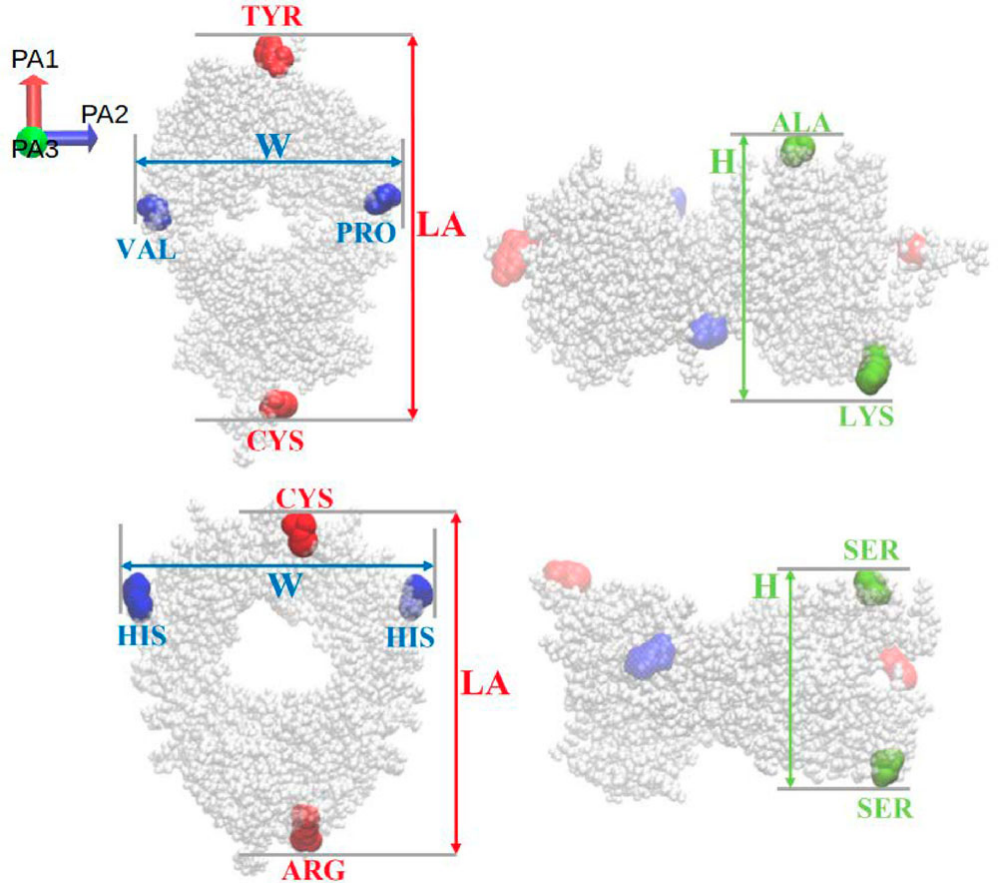
Definition of the long axis (LA), width (W) and height (H) of the Fab (top) and Fc (bottom) fragments. The coloured spheres show the residues used to calculate the protein dimensions. LA, W and H were obtained from the distance between the centers of mass of those residues.

The proteins investigated in this work feature an ellipsoidal shape, and therefore we can define a long axis (LA) and two perpendicular axes that quantify the width (W) and height (H) of the protein (see Figure 1). The maximum distance between the atomic pairs of selected residues (see Figure 1) along the long and two perpendicular axes was calculated for each frame in the trajectory. The component of these distances along the three principal axes (PA1 for LA, PA2 for W and PA3 for H) was used as the corresponding dimension.

### 2.4. Potential of mean force calculation

We used the umbrella sampling (US) [38] technique to compute the Fc-Fc and Fab-Fab potentials of mean force (PMF).

**Figure 2.**
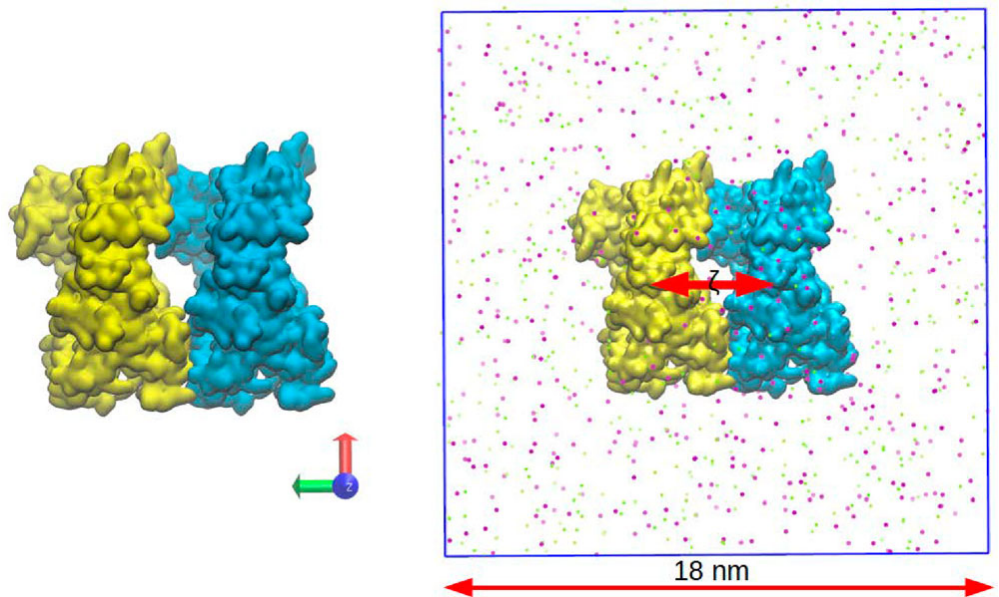
(Left) Initial conformation used for the Fc-Fc PMF calculation. (Right) Snapshot of the protein pair located in a periodic box. The Na ${}^{+}$ (green) and Cl ${}^{-}$ (purple) ions are also shown. Water is not shown for clarity.

The initial conformation of two Fc fragments is shown in Figure 2. The structure was generated and solvated in a box of dimension 18nm×18nm×18nm. 507 Na ${}^{+}$ ions and 511 Cl ${}^{-}$ ions were added to neutralise the charge of the two Fc fragments and attain a salt concentration of 150 mM. For the Fab-Fab system, 507 Na ${}^{+}$ ions and 529 Cl ${}^{-}$ ions were added. The whole system was minimised using the steepest descent method to remove bad contacts between the proteins, water and ions. The minimised system was equilibrated in the NPT ensemble for 5 ns with all the protein atoms restrained (spring constant $1000\;\mathrm{kJ}/(\mathrm{mol}\;{\mathrm{nm}}^{2})$) to their starting positions to equilibrate the system density and obtain an equilibrated ionic environment around the proteins. The pressure coupling was performed using the Berendsen barostat with a coupling constant of 0.5 ps. Following equilibration, the two proteins were pulled apart, by restraining the atoms belonging to one of the proteins to their starting positions while pulling the other away, at a velocity of 0.005 nm/ps, by restraining its center of mass with a harmonic potential of strength $1000\;\mathrm{kJ}/(\mathrm{mol}\;{\mathrm{nm}}^{2})$. From the pulling trajectory, we extracted a sequence of 51 configurations at increasing inter-protein distance, with a gap of 0.1 nm between consecutive configurations. These configurations were used as the starting structures for different umbrella windows. The distances in different windows were restrained to the desired values using a harmonic potential with force constant $1000\;\mathrm{kJ}/\mathrm{mol}/{\mathrm{nm}}^{2}$. We equilibrated the whole system to the desired pressure by using the Berendsen barostat with a coupling constant of 1 ps. The equilibration was performed for 5 ns in the NPT ensemble, keeping the center of mass of one protein fixed to its starting position, while the other protein was unrestrained and therefore free to rotate around its center of mass. Following equilibration, a minimum of 20 ns long NPT production runs were performed in each window with the Parrinello-Rahman barostat (coupling constant = 2.0 ps). During production, both proteins were free to rotate. The v-rescale [33] thermostat with a temperature coupling constant of 0.5 ps was used in all the free energy simulations. 51 windows were simulated with the interprotein distance varying between 3.0 nm to 8.0 nm. The PMF profile was reconstructed using the data obtained from the production runs using the GROMACS implementation of the WHAM algorithm [39].

### 2.5. Experimental methods

The Fab and Fc samples were provided by AstraZeneca as stock solutions in 25 mM Histidine buffer, 7 % w/v sucrose, pH 6.0. The samples were dialysed in the required buffer before use. The D ${}_{2}$O (99 % D), sodium chloride, phosphate salts and toluene were purchased from Sigma-Aldrich. The pure H ${}_{2}$O was obtained from a Millipore UHQ system at 18.2 MΩ · cm (Merck-Millipore, Watford, U.K.).

#### 2.5.1. Small angle neutron scattering (SANS) measurements

The radius of gyration (R ${}_{g}$) of the Fab and Fc domains was measured by small angle neutron scattering experiments performed using the instrument SANS2D (10.5286/ISIS.E.RB2010727-1) at ISIS Neutron Facility, RAL, STFC, UK. Fab and Fc samples were prepared in D ${}_{2}$O buffer (pH=7, IS=25mM, phosphate buffer) at the concentration of 2 mg/ml. The sample environment temperature is fixed at 25 ${}^{\circ }$C by a water bath system. The small angle neutron scattering intensity, I(q), was measured as a function of the momentum transfer, *q*. The momentum transfer, *q*, is determined by the neutron wavelength (*λ*) and scattering angle (*θ*) as $q=\frac{4\pi }{\lambda }\sin\theta$. Figure S2 (left) of SI shows the data for analysis after a standard data reduction and correction process. According to the Guinier approximation [40], the scattering intensity in the Guinier region ( $q\leq \frac{1.3}{R_{g}}$) can also be expressed as follows: $I(q)=I(0)\exp(\frac{-q^{2}R_{g}^{2}}{3})$, where R ${}_{g}$ is the radius of gyration, *q* is the momentum transfer, and I(0) is the zero-angle scattering intensity. Thus, R ${}_{g}$ can be directly determined from the Guinier plot of ln[I(q)] versus $q^{2}$, as shown in Figure S2 (right) of SI. A linear fit is made to the data in the Guinier region and the linear function's slope equals - $\frac{R_{g}^{2}}{3}$. While the ionic strength and buffer used in the experiments differ from the simulations, the R ${}_{g}$ of the Fab and Fc fragments, unlike the whole antibody, is not expected to depend strongly on the ionic strength. The ionic strength would, however, affect the inter-protein interaction and thus influence the R ${}_{g}$ of the whole antibody, as it depends on antibody conformation, which is determined ultimately by inter-fragment interaction.

#### 2.5.2. Static light scattering measurements

Static light scattering (SLS) was used to determine the 2 ${}^{nd}$ virial coefficients of Fab and Fc domains. The SLS measurements were taken by a Zetasizer Nano system (Malvern Instruments, Worcestershire, UK) following the method [41]. Different sample concentrations were prepared by dilution from the high-concentration sample stock. All samples were prepared in 150 mM NaCl solution at pH=7, and filtered through 0.22 *µ*m syringe filters before measurements. A UV spectrophotometer determined the concentrations of samples after filtration. A low-volume quartz cuvette (10 mm path length) was used for holding the sample. All measurements were conducted at 25 ${}^{\circ }$C. The 2 ${}^{nd}$ virial coefficient can be determined from the Debye plot as shown in Figure S3 of SI, in which $\frac{KC}{R_{\theta }}$ is plotted against the sample concentration, *C*. *K* is an optical constant, deduced by the light wavelength (633 nm) and $\frac{dn}{dC}$ value of mAb (0.185 mg/L). C is the sample concentration. R ${}_{\theta }$ is the Rayleigh ratio of scattered to incident light intensity, calibrated by measuring toluene as a reference. According to the Rayleigh scattering theory, the linear regression slope of the data points in the Debye plot equals twice the 2 ${}^{nd}$ virial coefficient. The DTS software (Malvern) was used for the data analysis.

## 3. Results and discussion

### 3.1. Protein structure

**Figure 3.**
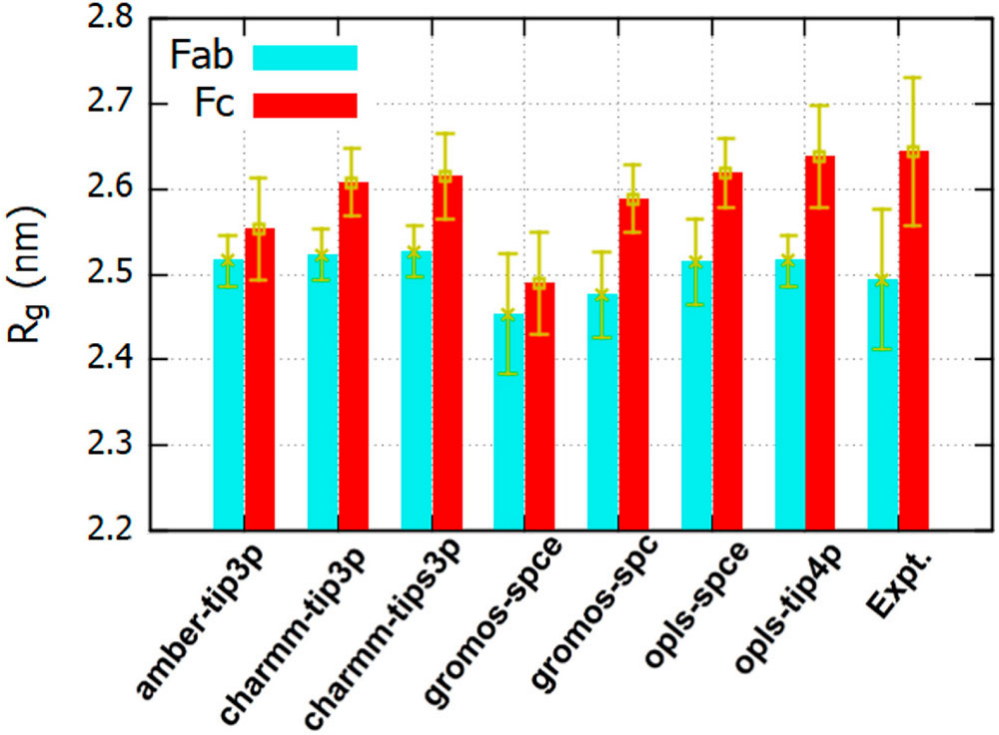
Radius of gyration for the Fab and Fc fragments for different ff/*wm*/ion parameter combinations.

**Figure 4.**
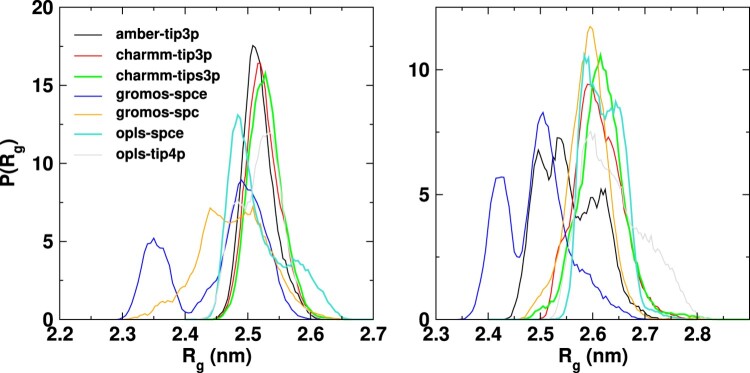
Probability distribution of the radius of gyration of Fab (left panel) and Fc (right panel) fragments for different ff/wm combinations. The average and standard deviations are listed in table S1 of SI.

**Figure 5.**
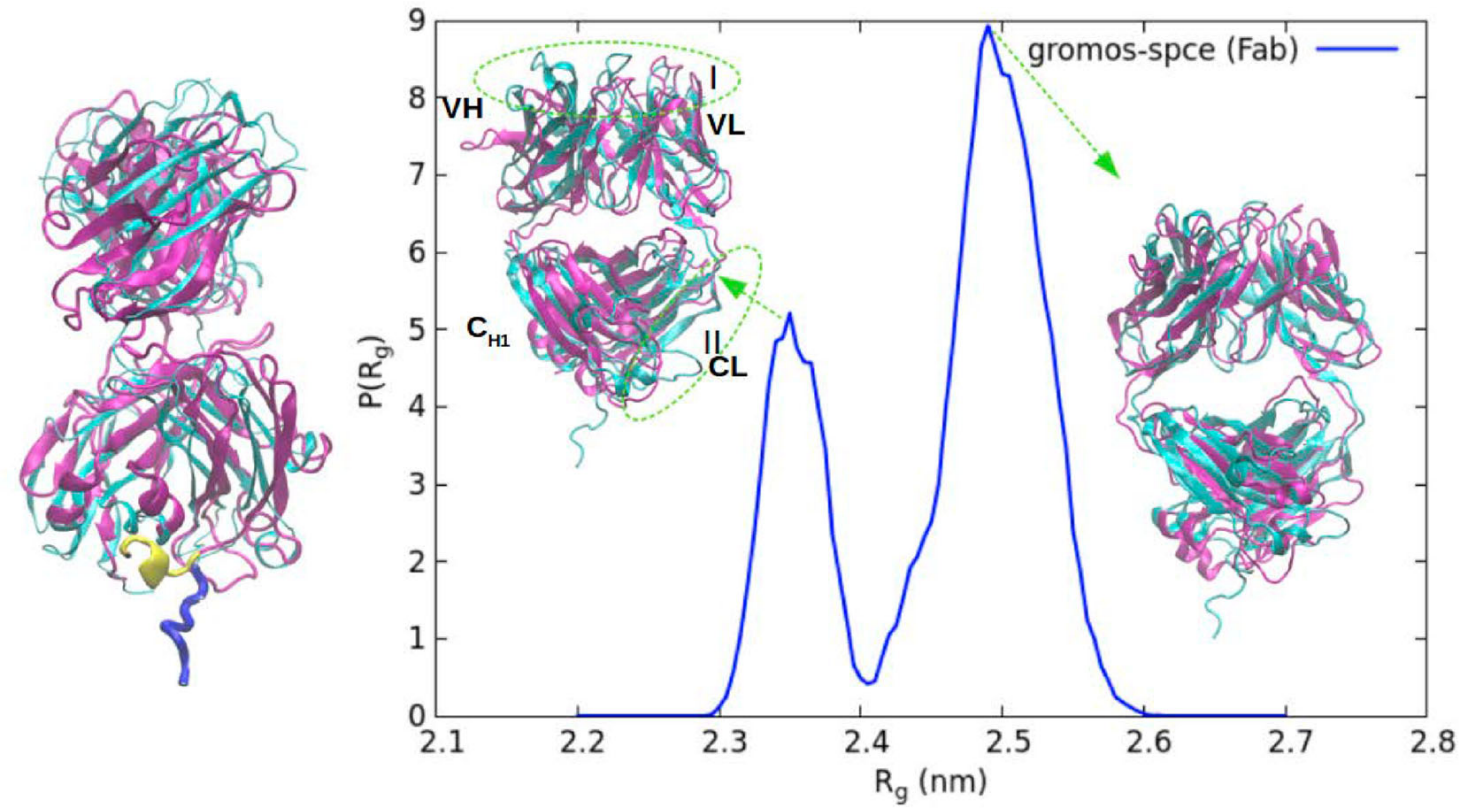
The structure of the Fab fragment corresponding to the two peaks of the R ${}_{g}$ distribution for the gromos-spce system, shown in purple, aligned with the minimised structure of the fab fragment (cyan). The regions that show deviation are highlighted as region I and region II. On the left is a snapshot of the Fab fragment corresponding to the peak at 2.35 nm, aligned with the minimised structure, showing a slight *α*-helical secondary structure in the terminal region.

The average radius of gyration ( $R_{g}$) of the Fab fragment in solution, varies between 2.45 and 2.53 nm (see Figure 3), hence showing a small dependence with the *ff* employed. Our results are comparable to the experimental value of 2.5 nm, determined from the SANS result using the Guinier approximation. The gromos *ff* features the largest deviation from the experimental data. The analysis of the probability distribution of R ${}_{g}$ shows that the gromos *ff* predicts two distinct peaks (see Figure 4). Further analysis of the gromos results (see Figure 5) indicate that the two peaks in the Fab correspond to two well defined conformations. The overlay of the simulated gromos structures (shown in purple) with the gromos minimised structure of the Fab fragment (shown in cyan), shows that in the structure corresponding to the lower maximum (2.35 nm), the loops in the target-binding region (region I in Figure 5) and near the C $H_{1}$ and CL domains of the Fab (region II) deviate significantly from the minimised structure. The loops in region I contain multiple TYR residues and drive the protein compaction. In addition, the C-terminal region of the Fab fragment features an intermittent *α*-helical conformation leading to a lower value of R ${}_{g}$ (see Figure 5). Advancing the discussion below, these results indicate that the gromos *ff* predicts higher protein hydrophobicity, which stabilises more compact conformations.

We find a stronger dependence of the R ${}_{g}$ of Fc on the *ff* employed, with average values spanning 2.49 nm and 2.64 nm (see Table S1 in the SI for numerical results). This change is significant, considering $R_{g}$ is a collective variable which is an effective average over different measures of protein dimension (like LA, W, H, see Figure 1). The radius predicted by the gromos *ff,* $R_{g}=2.59$ nm, which was parameterised with the spc water model (*wm*) to reproduce amino acid solvation free energies, is close to the results obtained with other state of the art *ff* s (charmm-tip3p), and with the value of 2.65 nm, obtained using SANS. We recall that unlike all-atom models, the gromos *ff* employed here does not include non-polar hydrogen atoms explicitly, and uses instead a united-atom representation for non-polar bond pairs. We performed additional simulations with the gromos *ff* and spce *wm*, as this model has been used in combination with the gromos *ff* in previous studies of proteins in solution [42]. Furthermore, the spce model outperforms spc in predicting structural and dynamic properties [43] and reproduces the solvation structure around amino acid analogues [44] better. The R ${}_{g}$ obtained with spce is significantly lower than the experimental one. This result shows that the *wm* influences the protein structure to a significant extent, and therefore the combination of protein *ff* s with different *wm*s must be exercised with care.

The average $R_{g}$ provides an important metric to assess the accuracy of *ff* s against experimental data. The probability distribution of $R_{g}$, on the other hand, provides information on the structural fluctuations of proteins in solution. We find that the probability distributions are not Gaussian (see Figure 4), showing evidence for competing structures both at low and high values of $R_{g}$. Generally, the gromos *ff* predicts much broader probability distributions for both Fab and Fc that are biased towards smaller values of R ${}_{g}$ and therefore a compact protein structure. We attach this behaviour to the inherently high combined protein solvation free energies arising from a combination of the free energy values of individual amino acids resulting from the gromos parameters. The gromos *ff* has been parameterised by fitting the solvation free energies of amino acid analogues to experiments. The solvation free energies determine the effective hydrophobicity of proteins. Previous gromos ff (53a6)-spce simulation of proteins at the water-vapour interface precdicted a strong protein adsorption. This behaviour has been attributed to the higher surface hydrophobicity of gromos proteins, which would be connected to the solvation free energy-matched gromos parameters [42]. Additional studies using gromos54a7 *ff* , pointed towards an enhanced protein aggregation propensity [45–47], which would be consistent with enhanced hydrophobicity.

**Figure 6.**
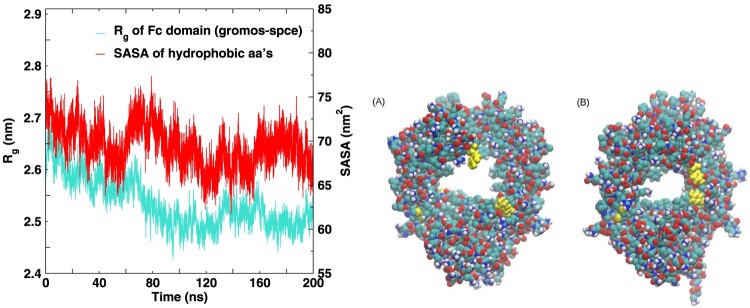
Time series of the R ${}_{g}$ (left y axis) of the Fc fragment for the gromos-spce system. The solvent accessible surface area (SASA) of the hydrophobic amino acids is also shown (see right y axis). The snapshots on the right depict the large amplitude internal motions in the  Fc fragment.

To gain further insight into the origin of the broad $R_{g}$ distributions predicted by the gromos *ff* for the Fc fragment, we analyzed the variation of $R_{g}$ with time (see Figure 6). $R_{g}$ decreases slowly with time, indicating a strong tendency for the proteins to form compact structures (see Figure 6). The slow dynamic rearrangement of the protein explains the broader probability distributions reported in Figure 4.

Microscopically, the monotonic decrease of R ${}_{g}$ can be traced back to the overly high hydrophobicity of the gromos protein surfaces. This enhanced hydrophobicity translates in a reduction of the contacts between hydrophobic amino acids and water. To support this notion, we calculated the total solvent accessible surface area (SASA) (see Figure 6) for the hydrophobic amino acids of the Fc fragment. The time dependence of SASA is clearly correlated with that of changes observed in R ${}_{g}$, supporting the idea that the observed reduction of R ${}_{g}$ with time is driven by a restructuring of the hydrophobic regions of the protein, leading to a shielding (since SASA decreases) of these regions from water. An example of such restructuring is shown in Figure 6. Two TYR residues in the Fc fragment that lie far away in the starting structure form intermittent contacts with each other resulting in large amplitude motions leading to a reduction of R ${}_{g}$ of the protein (see Figure 6). A similar large amplitude rearrangement was observed for amber ff, which results in a lower R ${}_{g}$ (and larger deviation from experiment) as compared to other *ff* s.

Overall, our results show that the simulated R ${}_{g}$ for Fc are systematically smaller than the experimental data, with larger deviations for gromos and amber *ff* s. Commonly used *ff* s are known to predict preferentially collapsed conformations for unstructured or flexible protein regions [48,49]. The Fc fragment features high flexibility in the hinge region. Hence, the inherent bias of the *ff* s towards collapsed conformations of such flexible regions may result in a smaller average value of R ${}_{g}$ that the *ff* s predict. The enhanced flexibility of the Fc fragment is consistent with earlier studies [50,51] of immunoglobulin G.

**Figure 7.**
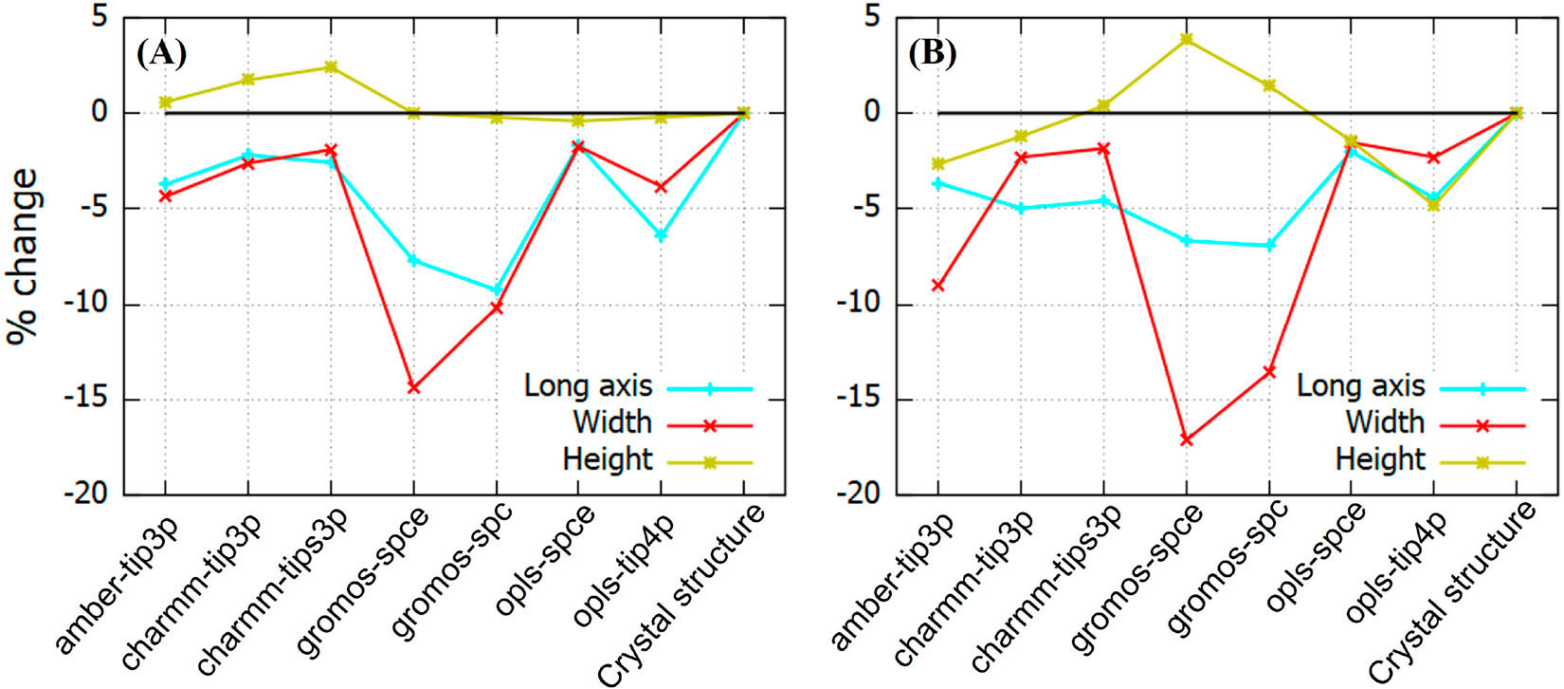
Deviations of the (A) Fab and (B) Fc dimensions, with respect to the crystal structure values, previously used in neutron reflectivity models [52].

While R ${}_{g}$ is a metric widely used to quantify the protein size, the Fab and Fc fragments feature nearly ellipsoidal shapes. A quantification of the full structure of these proteins is therefore important, as the protein anistropy must be taken into account e.g. while constructing models to interpret neutron reflectivity profiles (see e.g. [52,53]). We compile in Tables 3 and 4 the simulated dimensions of the proteins in solution. The dimensions in most cases, are not significantly different from those extracted from the crystal structure, although the width and long axis are systematically lower (see Figure 7). The gromos-spce combination predicts a considerably shorter long axis and width, both for the Fab and Fc fragments. For the Fab fragment, these shorter values are connected to the compact loop regions in the VH/VL domains (Region I in Figure 5) and the modification of the C ${}_{H1}$ and CL domains (region II in Figure 5). Similarly, the low values of the long axis and height for the Fc fragment originate from the structural modifications shown in Figure 6. From the snapshots of the Fc fragment shown in Figure 6, one can notice the reduction in width due to the structural changes. Similar rearrangement were observed with the amber *ff* , leading to shorter protein widths. Generally, the results for Fc show more variability with respect to the PDB reference data, reflecting the larger flexibility of Fc in solution.

**Table 3.** Dimensions of the Fab fragment for the different systems simulated in this work. The error bars were obtained from calculations performed with the three independent trajectories, for each system. The structure of the Fc domain was taken from pdb id. 1HZH, while the structure of the Fab domain was obtained from Ref. [17].

*ff*	*wm*	Long axis (nm)	Width (nm)	Height (nm)
amber99sb-ildn	tip3p	7.51 ± 0.2	5.50 ± 0.1	4.58 ± 0.01
charmm27	tip3p	7.63 ± 0.1	5.60 ± 0.2	4.63 ± 0.01
charmm27	tips3p	7.60 ± 0.1	5.64 ± 0.1	4.66 ± 0.04
gromos96 54a7	spce	7.20 ± 0.4	4.92 ± 0.1	4.55 ± 0.10
gromos96 54a7	spc	7.08 ± 0.3	5.17 ± 0.1	4.54 ± 0.10
opls/aa	spce	7.67 ± 0.3	5.74 ± 0.2	4.53 ± 0.02
opls/aa	tip4p	7.30 ± 0.1	5.53 ± 0.4	4.54 ± 0.05
Crystal Structure		7.8	5.75	4.55

**Table 4.** Dimensions of the Fc fragment for the different systems simulated in this work. The error bars are over the averages obtained from the three independent runs performed for each system.

*ff*	*wm*	Long axis (nm)	Width (nm)	Height (nm)
amber99sb-ildn	tip3p	7.38 ± 0.22	5.95 ± 0.30	4.05 ± 0.03
charmm27	tip3p	7.28 ± 0.10	6.39 ± 0.10	4.11 ± 0.02
charmm27	tips3p	7.31 ± 0.16	6.42 ± 0.10	4.18 ± 0.04
gromos96 54a7	spce	7.15 ± 0.10	5.42 ± 0.10	4.32 ± 0.26
gromos96 54a7	spc	7.13 ± 0.30	5.65 ± 0.18	4.22 ± 0.33
opls/aa	spce	7.50 ± 0.10	6.44 ± 0.03	4.10 ± 0.04
opls/aa	tip4p	7.32 ± 0.30	6.39 ± 0.16	3.96 ± 0.10
Crystal Structure		7.66	6.54	4.16

The results discussed above indicate that most of the *ff* s used in this study predict R ${}_{g}$'s close to the experimental value for the Fab fragment, except gromos *ff* which shows clear deviations when the simulations are performed with the spce *wm*. For the Fc fragment, we find a larger variability in the $R_{g}$ predictions, with gromos *ff* – spce predicting again larger deviations form the experiments followed by the amber *ff* . In case of the gromos *ff* , the observed deviations can be attributed to the enhanced amino acid hydrophobicity of this forcefield. The opls-tip4p and charmm-tips3p combinations predict R ${}_{g}$ in acceptable agreement with the experiments. These two *ff* s also predict protein dimensions similar to those extracted from PDB crystallographic structures.

### 3.2. Protein hydration and ionic structure

#### 3.2.1. Hydration structure

**Figure 8.**
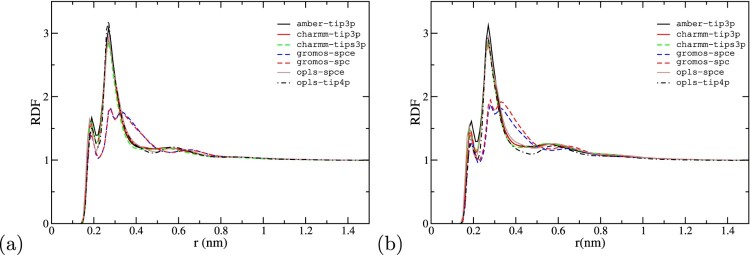
Radial distribution function of water around the (a) Fab and (b) Fc surfaces as a funciton of the distance (r) to the protein surface (r).

We analyzed the hydration structure around the protein by computing the un-normalised water-protein Radial Distribution functions (RDFs) using the code gmx rdf -surf included in the gromacs package. This code calculates the RDF by assigning each atom belonging to water to a bin corresponding to the minimum distance between the atom and the protein surface. The number of atoms in each distance bin is then divided by the bin-width. The integral of this RDF upto a distance *r* is, thus, the number of atomic pairs (protein-water) within distance *r* of the protein surface. We show the RDFs in Figure S4 of SI. To facilitate comparison between results obtained for different systems, we normalised the original RDFs by performing a linear fitting in the interval r=1−2nm. The RDFs were then divided by the corresponding linear fitting function. This rescaled plots (see Figure 8) show, more clearly, the water structuring as function of the distance to the protein surface.

As discussed above, the charge of Fab (+11e) and Fc (+2e) fragments at pH=7 is rather different. However, the hydration structure is very similar, (c.f. panels (a) and (b) in Figure 8), with a main peak located at 0.3 nm from the protein surface and very similar decay length, with the water structure approaching bulk behaviour at ∼1 nm. This decay length is very similar to the one reported before for water next to ionic surfactant layers [54] or hydrophobic surfaces [55]. The hydration structure, however, depends significantly on the *ff-wm* combination. The gromos *ff* , in combination with both spce and spc *wm*s, for instance, leads to a much weaker hydration of the protein surface as compared to other systems. This difference cannot be explained in terms of the *wm* only. Indeed, the hydration structure using spce and the opls *ff*, is much stronger, and similar to that obtained with other state of the art *ff* s, such as charmm with the tips3p water model. The weaker hydration is therefore connected to the weaker protein-water interactions and the fact that the amino acids modelled with the gromos *ff* are more hydrophobic. The larger hydrophobicity explains the tendency of the gromos proteins to adopt a more compact structure in water, which is consistent with the reduction in the level of hydration reported in Figure 8 (see also Figure S5 of SI). The ion-water distributions are shown in Figure S6 of SI and discussed in the associated text.

#### 3.2.2. Ion distribution around the protein

**Figure 9.**
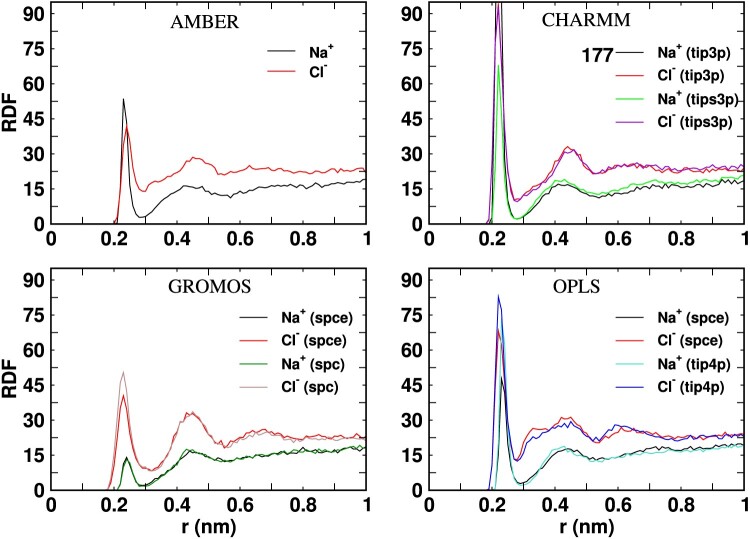
Radial distribution function of Na ${}^{+}$ and Cl ${}^{-}$ ions around the Fab surface as a functon of the ditance to the protein surface (r). The peak heights of the distributions not shown in full are indicated near the figure labels.

**Figure 10.**
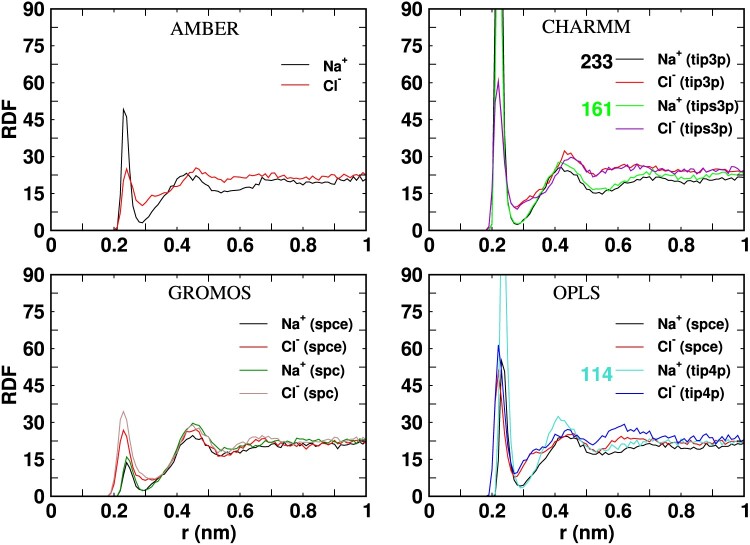
Radial distribution function of Na ${}^{+}$ and Cl ${}^{-}$ ions around the Fc surface as a function of the distance to the protein surface (r). The peak heights of the distributions not shown in full are indicated near the figure labels.

Ion-protein interactions play an essential role in regulating the protein-protein interactions, e.g. by adsorbing selectively at specific regions on the protein surface. Differences in the ionic environment around the proteins can lead to aggregation. Figures 9 and 10 shows the radial distribution functions (RDFs) of the ions as a function of the distance from the surfaces of the Fab or Fc fragments, using different *ff* combinations. The RDFs reveal apparent differences between the ionic distribution around the Fab and Fc. Firstly, the ionic double layer structure is weaker around the Fc fragment, as indicated by the larger accumulation of Cl ${}^{-}$ anions around the Fab fragment (c.f. RDFs in Figures 9 and 10). The stronger accumulation of anions around Fab is consistent with the higher positive charge of this protein (+11e), while Fc has a much lower net charge (+2e) at a pH of 7 (see Materials and methods). We note that the calculation of the ion-protein RDF converges in ∼10 ns, as shown in Figure S7 of SI.

The charmm *ff* predicts very strong ionic adsorption at the protein surface, as shown by the height of the first peak of the RDFs (see Figures 9 and 10). This effect is better seen in the radial coordination number plots (see Figure S8). The charmm *ff* predicts a higher, about 2−3 times, cation adsorption. Since the protein charge is the same, the strong adsorption of Na ${}^{+}$ and Cl ${}^{-}$ ions on the protein surface, for the different *ff* s, must be connected to the high value of $\epsilon_{vdW}$ employed in the charmm ff (see Table 2), which results in an effective reduction of the electrostatic repulsion of Na ${}^{+}$ from the positively charged protein surfaces. The stronger adsorption of charmm cations on the protein surface is reflected in the survival probability functions (see Section 8 of SI), which features much longer time decay, indicating a slowing down in the characteristic timescale defining the ion dissociation from the proteins.

The impact of the ionic vdW parameters can be investigated further by comparing the RDFs for the charmm and opls *ff* s. For the opls ff, the Cl ${}^{-}$ ions adsorb stronger than the Na ${}^{+}$ ions as the electrostatic interactions dominate due to weaker dispersion interactions of the opls Na ${}^{+}$ ions (see Table 2). We observe similar behaviour for the Fab fragment with the gromos *ff* , with stronger Cl ${}^{-}$ adsorption at the protein surface, originating from a small $\epsilon_{vdW}$ for the Na ${}^{+}$ ions and high $\epsilon_{vdW}$ for the Cl ${}^{-}$ ions combined with the electrostatic preference for the Cl ${}^{-}$ ions (see Table 2). These trends are consistent with the survival probabilities (see Figure S9 and S10 of SI), which indicate a faster decay of the time required for ion detachment in opls compared to gromos *ff* s.

The results discussed above show that the ionic environment around the proteins depends significantly on the *ff* employed, modifying the double layer structure and, potentially, the screening of the protein charge. To address the latter point, we calculated the net system charge ( $Q_{net}$) as a function of the distance from the protein surface:

(2) $$Q_{net}(r)=Q_{protein}+n_{+}(r)-n_{-}(r)$$

where $Q_{protein}$ is +11e for Fab and +2e for Fc, and $n_{\alpha }(r)$ is the number of ions with charge *α* within a distance *r* from the protein surface (see Figure S8 in the SI). The number of ions within a given distance *r* of the protein surface was obtained from the integral of the RDFs shown in Figures 9 and 10. The RDFs, as discussed before, represent the number of ions in a bin around the protein at a distance *r*, divided by the bin width. Thus, the cumulative number of ions up to a distance *r* around the protein is given by:

(3) $$n_{+/-}(r)=\int_{0}^{r}RDF_{Na^{+}/Cl^{-}}(r^{\prime })\;dr^{\prime }$$

**Figure 11.**
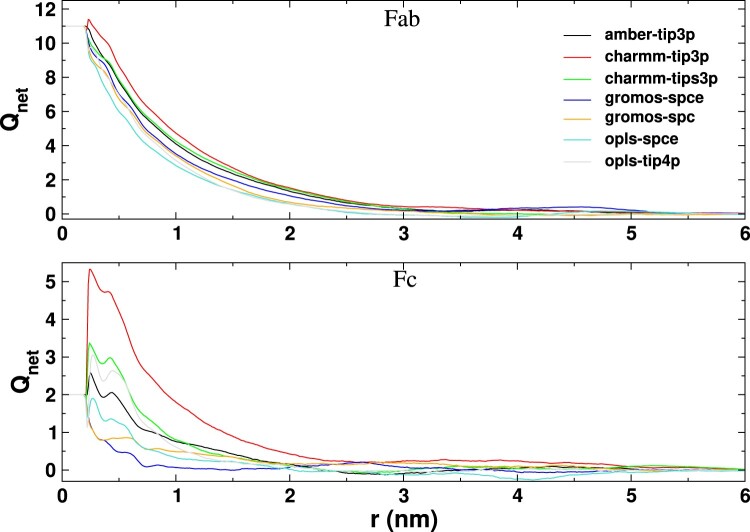
Net charge as a function of distance from the surface (r) of the Fab and Fc fragments for the different systems simulated in this work. The curves have been obtained for the last 100 ns of 200 ns long trajectories and have been further averaged over three independent runs.

Figure 11 shows the dependence of the accumulated charge as a function of distance from the Fab and Fc surfaces. For the Fab fragment, the charge compensation resembles the behavior based on simple meanfield electrostatics, namely a slow exponential decay characterised by the decay (Debye) length. For the Fc fragment, with positive net charge +2e, the charmm and opls *ff* s predict significant charge enhancement at the protein surface, revealing strong co-ion adsorption. This result shows that simple electrostatic arguments do not explain ion adsorption, and as noted above, dispersion interactions play an important role in determining the ion-protein interactions. Among all the *ff* s studies here, the charmm27-tip3p model leads to substantial charge enhancement, resulting in an effective surface charge ∼+5.5e. We find an increase for charmm27-tips3p too, but of different magnitude, corresponding to an effective surface charge of +3.3e (see Figure 11), hence a non-negligible difference. Our results show that the simulations performed with the tip3p or tips3p water models predict significantly different ion adsorption, particularly for the Fc fragment. This is a relevant result, as tip3p and tips3p have been used in the literature interchangeably [56,57]. Our results demonstrate that careful selection of an appropriate water model is required for modelling electrostatic properties.

**Table 5.** Debye length (*ζ*) obtained by fitting the $Q_{net}$ vs. *r* plots shown in Figure 11 with the function A ${}_{0}$e ${}^{-r/\zeta }$.

System	Fab (nm)	Fc (nm)	Dielectric constant
amber-tip3p	0.79 ± 0.01	0.62 ± 0.02	95
charmm-tip3p	0.83 ± 0.01	0.67 ± 0.01	95
charmm-tips3p	0.85 ± 0.01	0.56 ± 0.02	107
gromos-spce	0.72 ± 0.01	0.28 ± 0.01	73
gromos-spc	0.73 ± 0.01	1.07 ± 0.06	65
opls-spce	0.62 ± 0.01	0.45 ± 0.01	73
opls-tip4p	0.65 ± 0.01	0.54 ± 0.02	51

To quantify the decay length of the electrostatic interactions associated with the protein charge and the ion adsorption layers, we calculated the Debye length (*ζ*) for the different *ff* s investigated above. The decay of the net charge in Figure 11 can be fitted in most cases to an exponential function of the form A ${}_{0}$e ${}^{-r/\zeta }$, for radial distances in the region 0.25nm≤r≤1.0nm, which corresponds to the region of bulk ionic cloud around the protein surface. For the Fab fragment, the opls *ff* predicts the shortest *ζ* and the charmm *ff* the longest, with the values ranging from 0.62 nm to 0.85 nm. For the Fc fragment, we obtain a larger variability in the values of *ζ*, with values between 0.28 nm and 1.07 nm. This variability reflects deviations from the exponential decay of Q ${}_{net}$ with distance (see, e.g. the results from gromos-spce in Figure 11). As noted above, the ion-protein RDFs cannot be explained in terms of simple electrostatic arguments. Therefore, quantifying the charge decay length of Fc with distance, using *ζ*, is problematic. We report in Table 5 the values for this decay length as they might be helpful to inform future electrostatic models.

The Debye lengths calculated above can be compared with the results predicted by the Poisson-Boltzmann (PB) theory. The PB Debye length for monovalent 1:1 ions is defined by:

(4) $$\zeta ={\left(\frac{\epsilon kT}{2e^{2}N_{A}C}\right)}^{-\frac{1}{2}}$$

where, $\epsilon =\epsilon_{r}\epsilon_{0}$, $N_{A}$ is Avogadro's number, and *C* is the ionic concentration of the solution. For pure water, $\epsilon_{r}=78$, and the salt concentration employed here, C=0.15mol/L, we obtain ζ=0.79nm. This result is similar to computed decay lengths for the Fab fragment (see Table 5). Based on Equation (4) we expect some variability in *ζ* for different *ff* s, as the *wm*s feature different dielectric permittivities (see Table 5). Our data for Fab roughly follow the dependence predicted by Equation (4), with an increase of the Debye length with increasing dielectric permittivity (see Figure S11 in the SI).

#### 3.2.3. Protein-Protein potentials of mean force

The protein-protein interactions are often quantified experimentally using Static Light scattering (SLS), see e.g. [7,58]. The analysis of the SLS data using the Zimm method [59] gives access to the second virial coefficients, $B_{22}$, which quantify the inter-protein interactions, with negative B ${}_{22}$ indicating protein attraction, and positive B ${}_{22}$, repulsion. The virial coefficient can be calculated using the statistical mechanics equation:

(5) $$B_{22}=-\frac{2\pi N_{A}}{M_{w}^{2}}\int_{0}^{\infty }(g(r)-1)r^{2}\;dr$$

where $M_{w}$ is the molecular weight, 49635 g/mol [53], for the Fc fragment. Equation (5) assumes a protein with a spherical shape. Further, we approximate the radial distribution function using a low concentration approximation,

(6) $$g(r)=\exp\left(-\frac{\Delta G(r)}{RT}\right)$$

where ΔG(r) is the Gibbs free energy as a function of the the distance between the centers of mass of the proteins, *r*. We take *T* = 300 K consistent with our simulation conditions.

**Figure 12.**
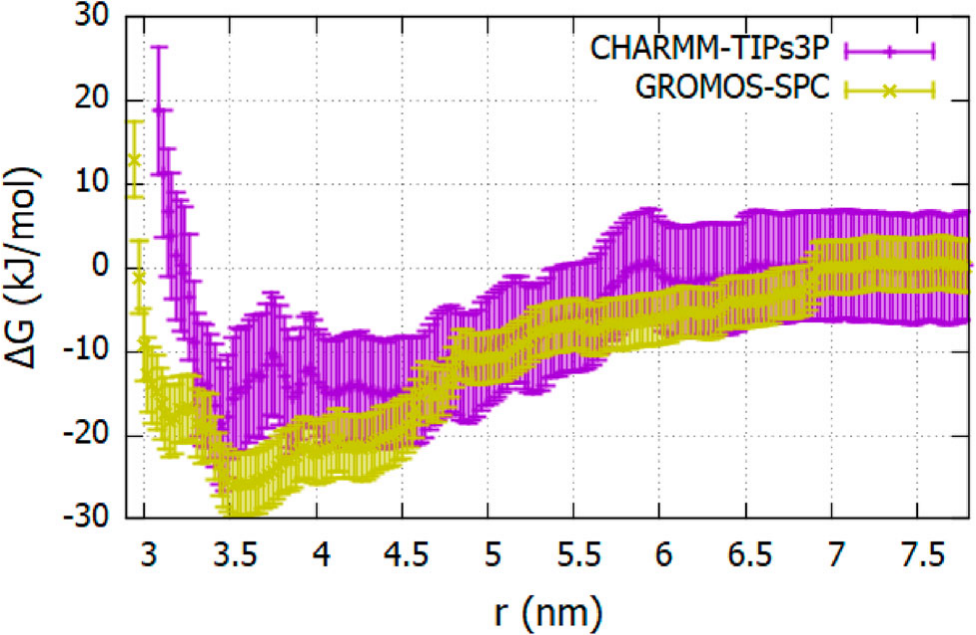
The Fc-Fc ΔG(r) profiles calculated using the charmm27 (see Figure S12 of SI for additional details) and gromos96 54a7 as a function of the center of mass separation between proteins (see Figure S13 of SI additional details) *ff* s.

We computed the Fc-Fc potential of mean force using charmm-tips3p and gromos-spc parameters (see Figure 12). These calculations provide a route to assess the accuracy of different *ff* s in reproducing the interaction of therapeutic proteins in solution. For Fc fragment, we calculated B ${}_{22}$ from two independent umbrella sampling trajectories using the charmm-tip3p forcefield (see Figure S12 and S13 in the SI for results obtained using these trajectories). We used the same ‘reaction’ coordinate for both trajectories. Figure S12 shows that the PMFs can differ significantly, even though there are no significant deviations in the relative orientations of the proteins generated with these trajectories (see snapshots in Figure S13). The different PMFs highlight the rather complex sampling space of the protein-protein interactions. For subsequent analysis, we used the PMF corresponding to the stronger attraction. It is expected that in a restraint-free environment, the proteins will freely explore different conformations, and the most likely conformation will be the one corresponding to more attractive PMF.

Figure 12 shows the PMF corresponding to the strongest Fc-Fc attraction obtained with the charmm-tips3p model. We compare this result with the PMF from gromos-spc parameters obtained using a single trajectory. The inter-protein interactions vary significantly with the *ff* . The charmm *ff* predicts weaker attractive interactions, with a binding energy of ∼−14kJ/mol. The gromos *ff* predicts a considerably stronger attraction with a binding energy of ∼−27kJ/mol. This strong attraction is consistent with the enhanced amino acid hydrophobicity discussed above, which drives stronger inter-protein attraction. The enhanced hydrophobicity is also reflected in the water coordination number for the proteins, which are significantly lower than those obtained with other state-of-the-art *ff* s (see Figure S5 of SI). Our results reveal a clear correlation between hydration level and inter-protein interaction.

We calculated the virial coefficient using the Equations (5)-(6) for the PMFs shown in Figure 12. For charmm-tip3p system we obtain a slightly negative coefficient, $B_{22,charmm-tips3p}=-1.34\times 10^{-2}\;\mathrm{mol}\;\mathrm{mL}/g^{2}$. For the second charmm trajectory (corresponding to weaker PMF, see Figure S11), the virial coefficient is slightly positive $1.2\times 10^{-4}\;\mathrm{mol}\;\mathrm{mL}/g^{2}$. This virial coefficient is similar in magnitude to that reported for lysozyme at pH ∼7, 100 mM electrolyte concentration and 1 mg/mL solutions [58]. The Gromos *ff* predicts very large virial coefficients, $B_{22,gromos-spc}=-26.28\times 10^{-2}\;\mathrm{mol}\;\mathrm{mL}/g^{2}$. This value is very high, reflecting the enhanced hydrophobic character of the gromos Fc fragment. We note that such large virial coefficients have been reported before in molecular simulation studies using coarse-grained models (MARTINI) of lysozyme [60], and hence they are not unusual in simulation studies. Those authors showed that reducing the protein-protein dispersion interactions provided a better description of the experimental data. This notion aligns well with our conclusion that the gromos *ff* used here over-predicts the amino acid interaction, resulting from an enhanced protein hydrophobicity and consequently, strong protein-protein interactions, which is reflected in the large negative virial coefficient, $B_{22,gromos-spc}$.

**Figure 13.**
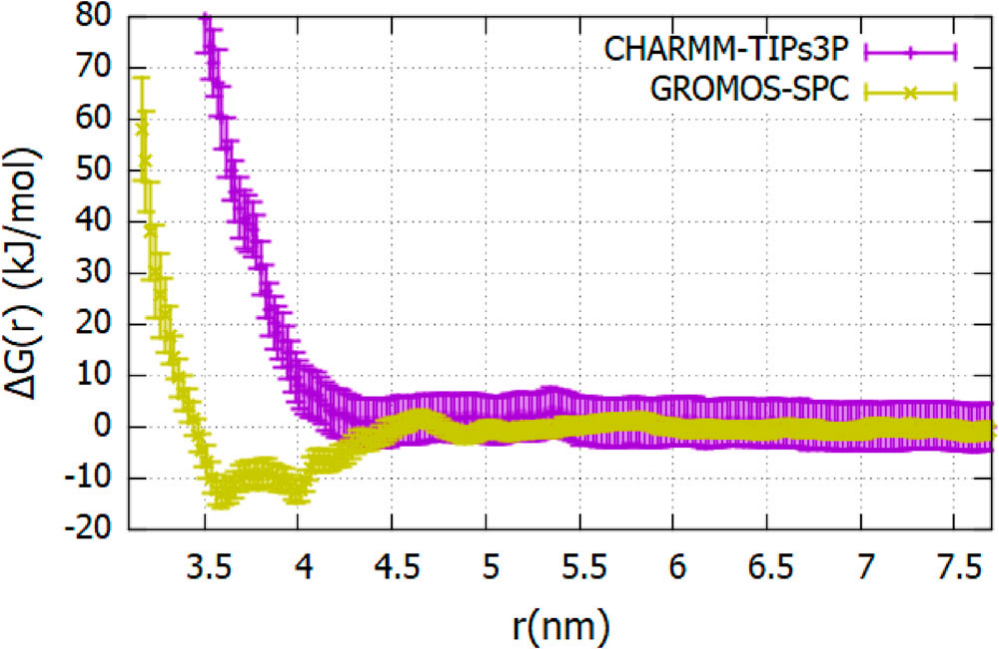
The Fab-Fab ΔG(r) profiles calculated using the charmm27 and gromos96 54a7 *ff* s as a function of the center of mass separation between proteins.

The umbrella sampling trajectory generated with the charmm forcefield predicts a Fab-Fab PMF that is mostly repulsive (see Figure 13) at short inter-protein distances (3.5–4.5 nm). In contrast, gromos predicts a deep attractive minimum in this distance range. The stronger attraction is consistent with the higher hydrophobicity of the gromos proteins in water. The virial coefficients (using $M_{w,Fab}=47450\;g/\mathrm{mol}$), are: $B_{22,charmm-tips3p}=0.8\times 10^{-4}\;\mathrm{mol}\;\mathrm{mL}/g^{2}$ (repulsive interactions), and $B_{22,gromos-spc}=-10.0\times 10^{-4}\;\mathrm{mol}\mathrm{mL}/g^{2}$ (attractive interactions). These results again demonstrate that the protein-protein interactions depend significantly on the *ff* parameters.

To assess the accuracy of the forcefields discussed above, we performed Static Light Scattering experiments at conditions consistent with the molecular dynamics simulations. The experimental virial coefficients are listed in Table 6, along with the simulated results. The experiments show a strong attraction between Fc and repulsion between Fab. The large magnitude of the experimental values might be connected to the lack of buffer in the solution. It is known that the buffer influences the magnitude of the virial coefficients and protein stability against aggregation (see Ref. [7]). While Table 6 shows substantial deviations between the simulated and experimental results, the charmm forcefield follows the general experimental trends, which is attraction between two Fc fragments and repulsion between Fabs. However, the gromos *ff* predicts a virial coefficient for Fc that is already much more negative compared to the experimental results. Hence, we conclude that the gromos *ff* predicts an unusually strong attraction between these antibody fragments. The convergence of the PMF profiles for the 20 ns production runs performed in each umbrella window performed to calculate the PMFs is shown in Figure S14 of SI.

**Table 6.** Table of B ${}_{22}$ values from simulations and experiments. The results were obtained using the data shown in Figures 12 and 13.

	charmm-tips3p	gromos-spc	Experiments
Fc	$-1.34\times 10^{-2}$	$-26.28\times 10^{-2}$	$(-7.77\pm 1.55)\times 10^{-2}$
Fab	$0.08\times 10^{-3}$	$-0.1\times 10^{-2}$	$(3.275\pm 0.93)\times 10^{-3}$

## 4. Conclusion and discussion

We have investigated the hydration structure, ion-protein interaction, and protein-protein interaction of the Fab and Fc fragments of mAb COE3, using state-of-the-art atomistic *ff* s (charmm, amber, opls and gromos) and molecular dynamics simulations.

All the *ff* s predict similar radii of gyration for the Fab fragment. We find that this fragment features small flexibility; therefore, the average structure in the solution is very well-defined. In contrast, the Fc fragment is highly flexible, especially near the hinge region, and the radii of gyration predicted by the different *ff* s differ from each other. charmm and opls are closer in agreement with experiments, while the gromos *ff* , and to some extent, the amber *ff* underestimate the protein size. The smaller size predicted by the gromos *ff* can be traced back to the enhanced hydrophobicity of the proteins, leading to more compact proteins. The protein size is correlated with the hydration strength, and *ff* s that over-predict hydrophobicity feature significantly weaker solvation shells.

The MD simulation trajectories provide information that can be helpful to interpret experimental results obtained, *e.g.* using neutron reflectivity. Fab and Fc are anisotropic proteins whose shape can be described by three perpendicular axes that quantify protein anisotropy. Our estimates of the protein dimensions in solution agree reasonably well with the results employed in previous experiments, which were based on crystal structure data. For most *ff* s, the deviations vary between 5–10%, with the gromos *ff* showing more significant differences, up to 15%, from the crystal data. Computationally derived protein dimensions consider the protein's fluctuation in solution. These predictions might provide an approach to improve the models needed to deconvolute the information obtained from neutron experiments.

It is known that state-of-the-art *ff* s might predict compact structures, particularly in the disordered protein regions. We found that the protein compactness can also depend on the *wm* employed. Specifically, we find that the combination of the gromos and spce *ff* results in very compact proteins. This *ff* combination is popular in the simulation community. Our results indicate that such an approach should be used carefully when modelling flexible proteins, such as the Fc fragment.

The ionic double layer structure around the highly charged Fab protein (+11e at pH=7) resembles the structure expected based on simple mean field electrostatic theories. Namely, a build-up of counterions in the neighbourhood of the protein surface and an approximately exponential decay of the charge density from the protein surface. However, we also find evidence for deviations from the simple electrostatic picture. These are more evident for Fc, which has a lower charge (+2e at pH=7). In this case, we found an enhancement of the protein charge modelled with the charmm *ff* and tip3p or tips3p *wm*s. The enhancement emerges from the accumulation of co-ions (Na ${}^{+}$) on the protein surface, with a higher enhancement when the tips3p *wm* is employed. We, therefore, show that the double layer structure can depend significantly on the *wm* employed, particularly when considering proteins with low charge. This result is significant since the *wm*s, tip3p and tips3p, have been used interchangeably in previous works. Our results show that differences can be expected when considering ion-water interactions. Beyond the relevance of our work to the specific modelling issues of these complex proteins, our simulations point towards the importance of non-electrostatic dispersion interactions in determining ion adsorption and, therefore, the structure of the ionic cloud around the proteins. The latter is expected to play an essential role in defining protein-protein interactions.

Regarding inter−protein interactions, we found that interaction strength depends considerably on the *ff* parameters. The gromos *ff* predicts a strong, attractive interaction between Fc fragments ( ∼30kJ/mol). The state-of-the-art *ff* charmm indicates a weaker attraction ( ∼14kJ/mol) with tips3p. These differences in attractive interactions result in disparate virial coefficients. We obtained a very large negative value with the gromos *ff* indicating strong protein interactions. charmm predicts slightly negative viral coefficients, with values consistent in magnitude, with those reported in experiments of smaller proteins (lysozyme). Regarding the Fab fragments, the charmm virial coefficient is positive, indicating repulsion, compatible with the high electrostatic charge of Fab (+11e) at pH=7. Our Static light scattering experiments show that Fc features a strong negative virial coefficient in the absence of buffer, indicating strong protein attraction. However, Fab features a positive virial coefficient indicating protein repulsion. This experimental behaviour is reproduced by the charmm *ff* , while gromos predicts strong attraction (negative virial coefficients) for Fab and Fc fragments. These results support the idea that gromos proteins have a stronger hydrophobic character. We note, however, that the computation of virial coefficients for these complex proteins is challenging due to the configurational space determining the potential of mean force. To calculate our potentials of mean force, we chose similar reaction coordinates for the different systems to ensure a consistent comparison of the results obtained with different models. It will be interesting to expand our investigation by using *e.g.* enhanced sampling techniques to compute PMFs along other reaction coordinates, which should allow the quantification of the virial coefficients taking into account non-spherical protein geometries. Albeit more challenging, the PMF computations using the methods discussed in this work, might be extended to other fragment pairs like Fab-Fc, as well as full antibodies, taking advantage of more advanced enhanced sampling techniques.

Overall, our results indicate that the simulation of large proteins, such as monoclonal antibodies, must be performed with care. As shown here, different state-of-the-art *ff* s predict significant differences in the solvation, ion adsorption (double layer structure) and inter-protein interaction. More experimental results are needed to develop accurate *ff* s to investigate the microscopic behavior of these complex therapeutic proteins.

## Supplementary Material

Supplemental Material
