# Supplementary material for: Structure and interaction of therapeutic proteins in solution: a combined simulation and experimental study
Source: Mol Phys. 2023 Jul 17;121(19-20):e2236248. doi: 10.1080/00268976.2023.2236248 (PMC10721229; doi:10.1080/00268976.2023.2236248)
Supplement: Supplemental Material [file TMPH_A_2236248_SM6847.pdf]

Structure and interaction of therapeutic proteins in solution: A combined simulation and experimental study
Supplementary Information

Suman Saurabh[a], Zongyi Li[b], Peter Hollowell[b], Thomas Waigh[b, d], Peixun Li[e], John Webster[e], John M. Seddon[a], Cavan Kalonia[c,*], Jian R. Lu[b,*] and Fernando Bresme[a,*]

[a]Department of Chemistry, Molecular Sciences Research Hub Imperial College, W12 0BZ, London, United Kingdom; [b]Biological Physics Group, School of Physics and Astronomy, Faculty of Science and Engineering, Oxford Road, The University of Manchester, Manchester M13 9PL, UK; [c]Dosage Form Design and Development, BioPharmaceutical Development, BioPharmaceuticals R&D, AstraZeneca, Gaithersburg, Maryland 20878, United States; [d]Photon Science Institute, The University of Manchester, M13 9PL, UK; [e]STFC ISIS Facility, Rutherford Appleton Laboratory, Didcot, OX11 0QX, UK

ARTICLE HISTORY
Compiled June 26, 2023

CONTACT A. N. Author. Email: cavan.kalonia@astrazeneca.com
CONTACT A. N. Author. Email: j.lu@manchester.ac.uk
CONTACT A. N. Author. Email: f.bresme@imperial.ac.uk

1. Structure of the Fab and Fc domains

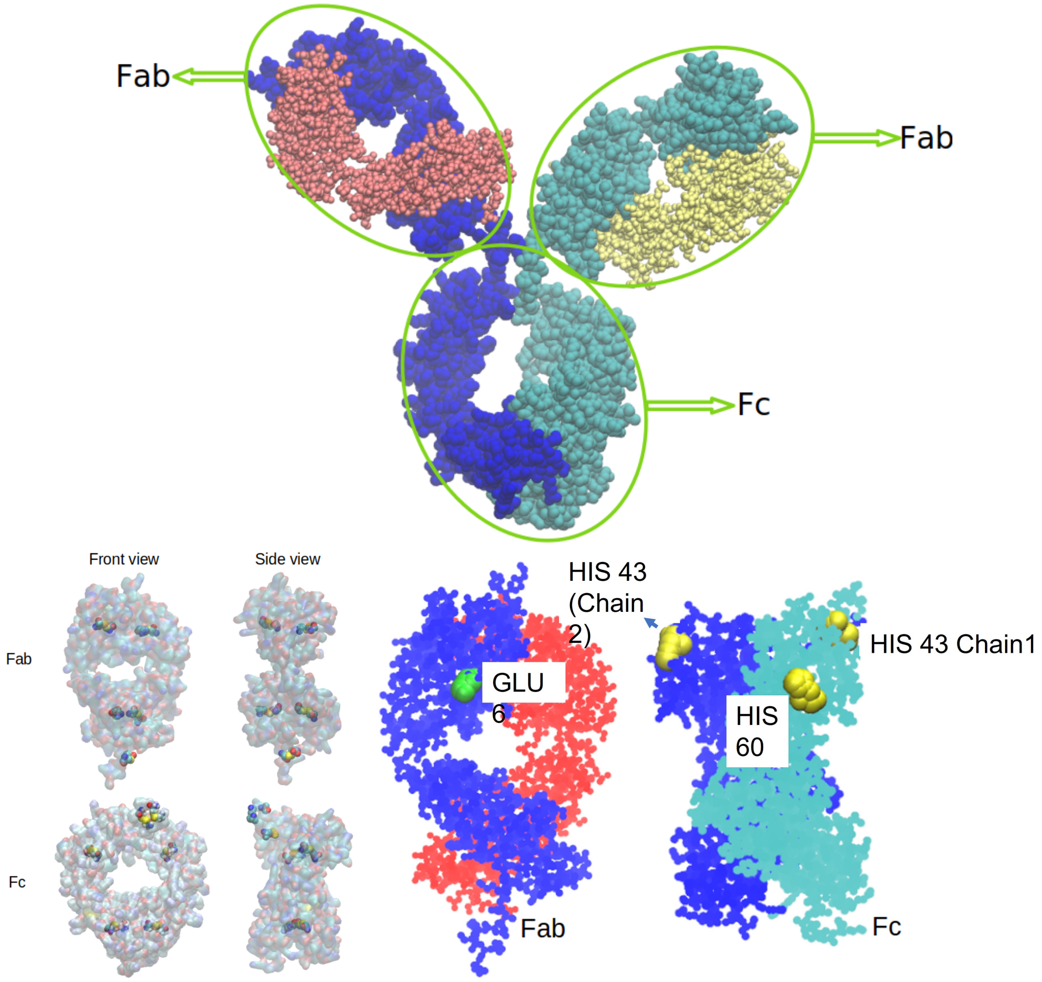

Figure S1.: **(Top) The crystal structure of mAb COE3. The two heavy chain are shown in blue and cyan, while the two light chains are shown in red and yellow. The regions constituting the Fab and Fc domains are shown. (Bottom, left) The Front and side-views of the Fab and Fc domains are shown along with the position of the disulphide bonds. (Bottom, right) On the right are shown the Fab and Fc fragments and the GLU (green) and HIS (yellow) residues that were protonated at pH = 7.**

2. Experimental results

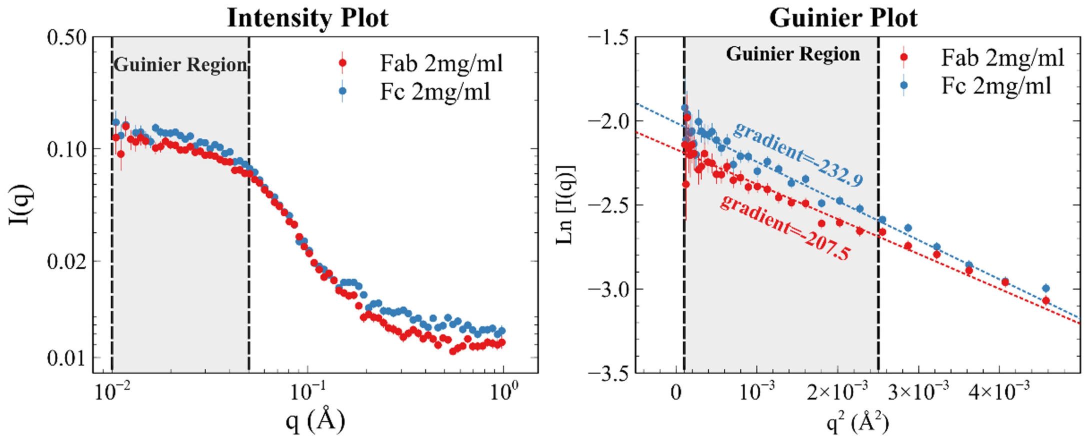

Figure S2.: **SANS results of Fab (red) and Fc (blue) were measured at the concentration of 2mg/ml and pH 7. The Guinier region was indicated with a grey background. (Left) The intensity plot of the collected data covers the whole q range measured. (Right) the Guinier analysis of the SANS data. The dashed lines show the linearization of the data points in the Guinier region; the slopes of the linear fits were labelled.**

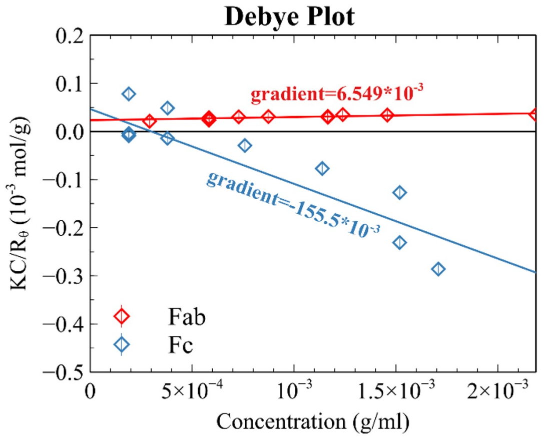

Figure S3.: **Debye Plot of the SLS results of Fab (red) and Fc (blue) in 150mM NaCl solution at pH 7. The scatter plots show the measurements at different sample concentrations. The solid line shows the linear regression fitting.**

3. Numerical values of R_g of the Fab and Fc domain.

Table S1.: **Radius of gyration (in nm) of the Fab and Fc domains for the different systems simulated in this work. The error bars are over the averages obtained form the three independent runs performed for each system.**

force field	Fab	Fab (last 120 ns)	Fc	Fc (last 120ns)
amber-tip3p	2.52 ± 0.03	2.51 ± 0.003	2.55 ± 0.06	2.57 ± 0.07
charmm27-tip3p	2.52 ± 0.03	2.51 ± 0.01	2.61 ± 0.04	2.60 ± 0.02
charmm27-tips3p	2.53 ± 0.03	2.52 ± 0.03	2.61 ± 0.05	2.61 ± 0.02
gromos96 54a7-spce	2.45 ± 0.07	2.49 ± 0.07	2.49 ± 0.06	2.47 ± 0.04
gromos96 54a7-spc	2.48 ± 0.05	2.47 ± 0.03	2.59 ± 0.04	2.58 ± 0.01
opls/aa-spce	2.52 ± 0.05	2.51 ± 0.03	2.62 ± 0.04	2.61 ± 0.03
opls/aa-tip4p	2.52 ± 0.03	2.52 ± 0.02	2.64 ± 0.06	2.62 ± 0.04

4. Protein-water RDFs

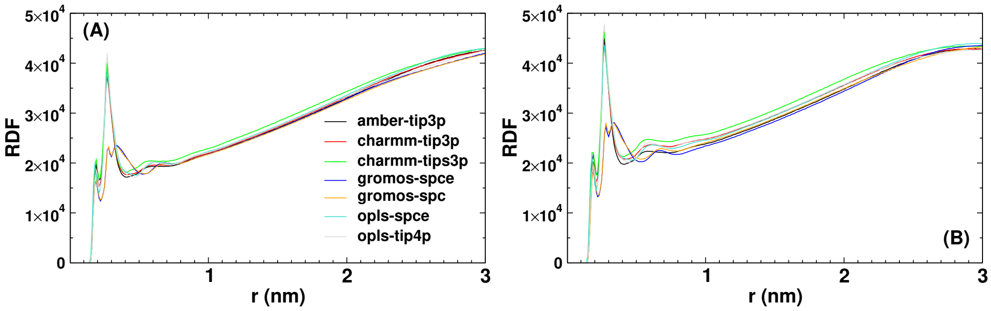

Figure S4.: **Radial distribution function of water around the (A) Fab and (B) Fc surfaces.**

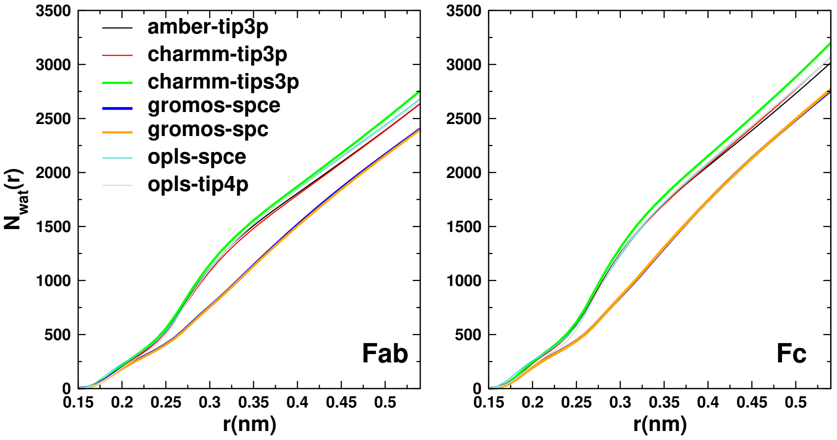

Figure S5.: **Number of water molecules within a distance r of the Fab and Fc surface.**

5. Ion-water Interaction

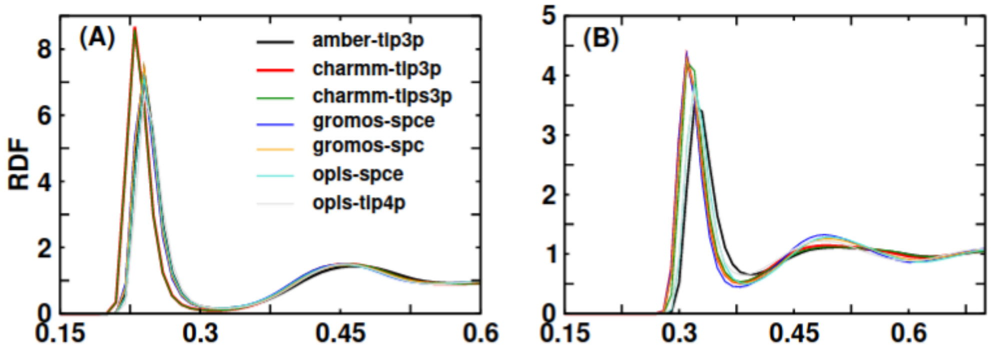

Figure S6.: **The radial distribution functions for the water oxygens around the (A) Na^+ and (B) Cl^- ions.**

To understand the different ionic adsorption predicted by the charmm forcefield using the TIP3P or TIPs3P water models and gromos using spc or spc/e water, we calculated the ion-water radial distribution functions (RDFs). Specifically, we calculated the radial distribution function for the water oxygen around the Na^+ and Cl^- ions. Our results reveal signifcant differences between the RDFs around Na^+ obtained for the charmm27 systems and all the other systems. The Na^+-water interactions were stonger in case of the charmm27 systems. Similar to the Na^+ ions, in case of the Cl^- ions the distributions for the different systems fall into two groups. The charmm and gromos systems for both water models used (tip3p and tips3p for the charmm system and spce and spc for the gromos systems), depict a stronger interaction between the Cl^- ions and water as compared to the other systems.

6. Convergence of RDF data

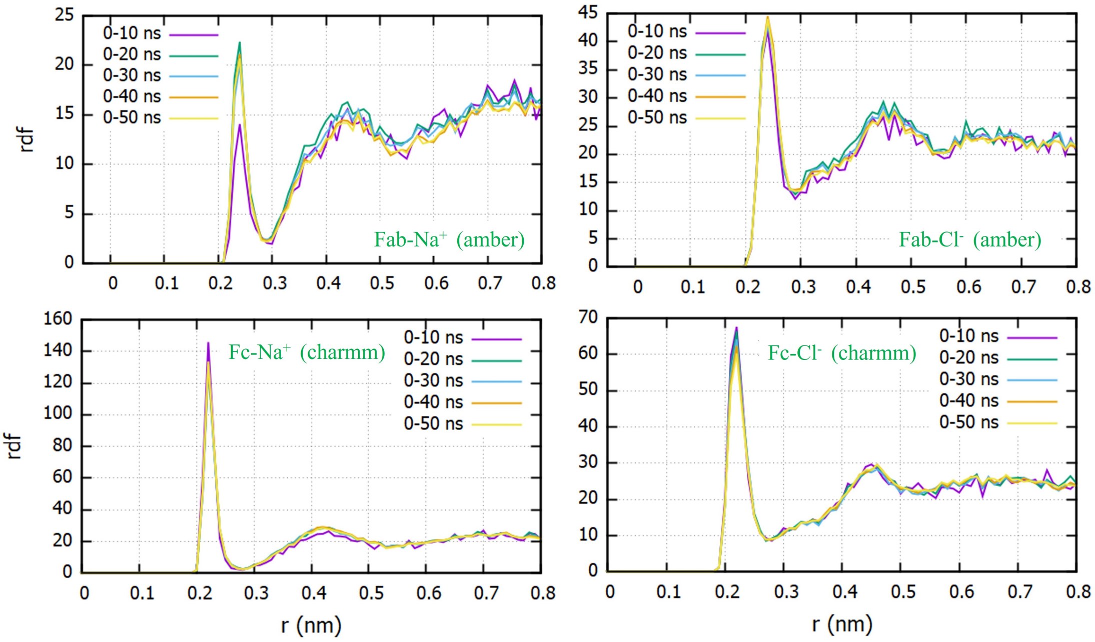

Figure S7.: **The radial distribution function for different time intervals. The plots illustrate the convergence of the rdf calculation.**

7. Co-ordination numbers

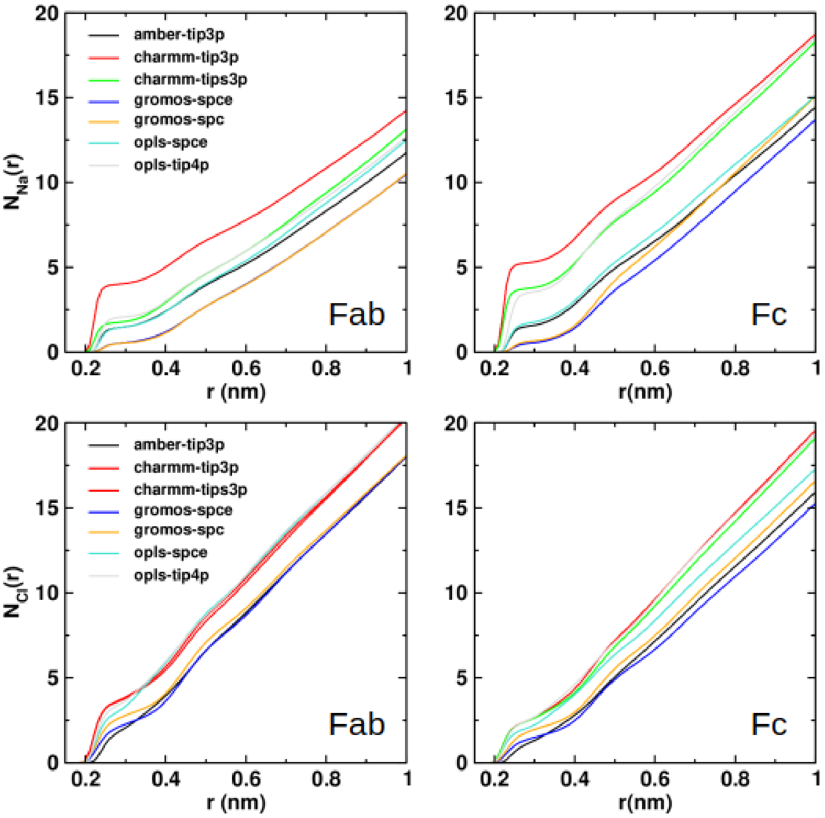

Figure S8.: **Number of ions within a distance r of the Fab and Fc surface.**

8. Ion kinetics

8.1. Survival probability calculation for Protein-Ion contacts

We computed the survival probability function of protein-ion contacts by analyzing the average time required for detachment of an ion from the protein surface. This approach requires defining a distance threshold for protein-ion attachment, which was defined as the shortest of all protein-ion pair distances, d_{min}, which is the minimum of all the distances between an ion and the atoms of the protein. An ion was deemed to be adsorbed on the protein if its $d_{min} \leq 0.4$ nm (r_{cut}).

The time series of d_{min} was calculated for each ion. We show in Figure S9 the variation of d_{min} with time for a single ion. The trajectory can be decomposed into a series of intervals: time regions where the d_{min} for an ion either lies within or beyond a distance of r_{cut} from the protein surface. The stretch of time for which $d_{min} \leq r_{cut}$ corresponds to a *residence event* and the time interval is called the *residence time* (τ_r). The time dependence of d_{min} for all ions was used to study the adsorption kinetics by

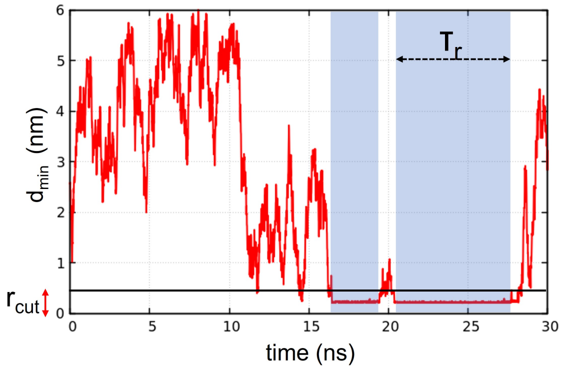

Figure S9.: **Time dependence of the minimum distance, d_{min}, of an ion from the Fc surface. The regions of the trajectory where d_{min} is below $r_{cut} = 0.4$ nm correspond to adsorption events (shaded in green) while the rest of the trajectory corresponds to free diffusion of Na^+ ion in solution. The length of an adsorption event τ_r defines a residence time.**

calculating the survival probability $S(t)$,

$$S(t') = \frac{\langle h(0)h(t')\rangle}{\langle h(0)h(0)\rangle} \tag{1}$$

where $h(0) = 1$ if a protein-ion contact is present at time $t = 0$ and, $h(0) = 0$ if there is no contact. Similarly, $h(t') = 1$ if a contact initially present at t=0, is still intact at time $t = t'$. If re-attachment takes place due to diffusion of a given ion back from the solution, we consider this event as a new adsorption event. $S(t)$ can thus be defined as the probability that an ion-protein contact that exists at time 0, continues to exist at least up to time t.

8.2. Plots of survival probability

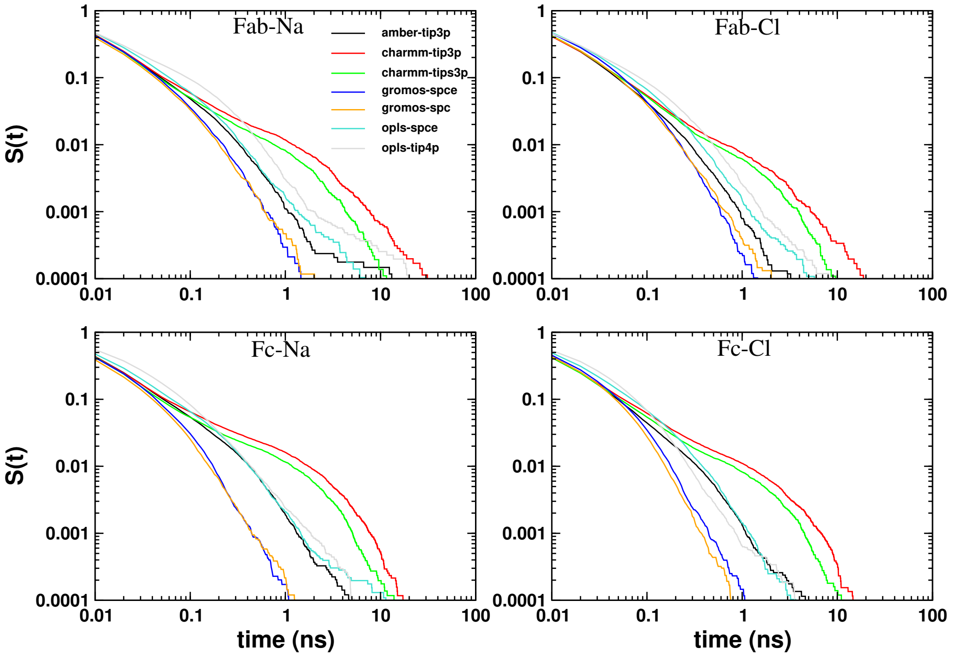

Figure S10.: **The survival probabilities of the protein-ion contacts.**

We have demonstrated that the protein-ion interaction can lead to strong adsorption of the ions on the protein surface (as seen in the height of the contact peaks shown in figures 9 and 10 of the main text). To gain further insight into the the relative ion-protein interactions for the different force fields, we calculated the survival probability of ion-protein contacts as discussed in section 8.1 above. Figure S10 shows the survival probability functions for Na^+ and Cl^-interacting with Fab or Fc. The log−log plot shows different regimes, which cannot be fit with a single exponential. This behavior agrees with our previous analysis of the survival probability of histidine at mAb surfaces [1], and it follows the non-Markovian process observed in the detachment dynamics of water.

References

[1] S. Saurabh, C. Kalonia, Z. Li, P. Hollowell, T. Waigh, P. Li, J. Webster, J.M. Seddon, J.R. Lu and F. Bresme, Molecular Pharmaceutics **19** (9), 3288–3303 (2022).

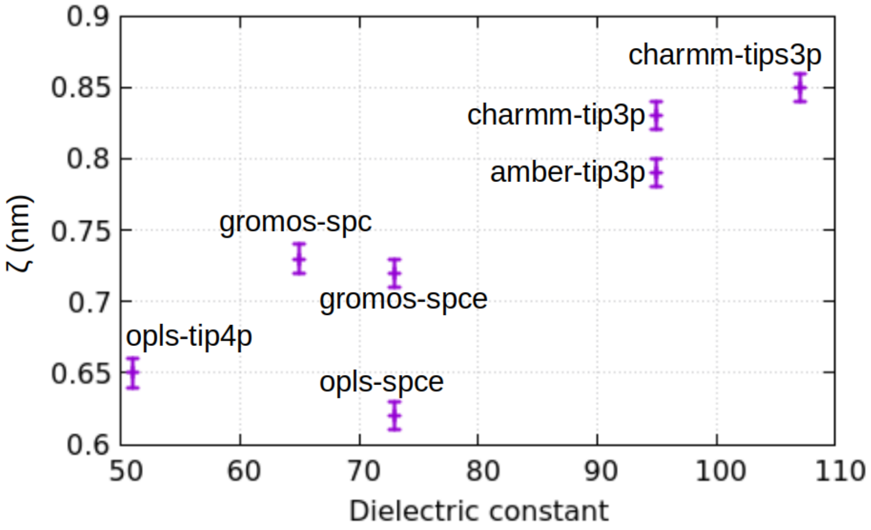

Figure S11.: **The variation of Debye length (ζ) for different systems as a function of the dielectric constant of the water model used.**

9. Fc-Fc conformations for umbrella windows

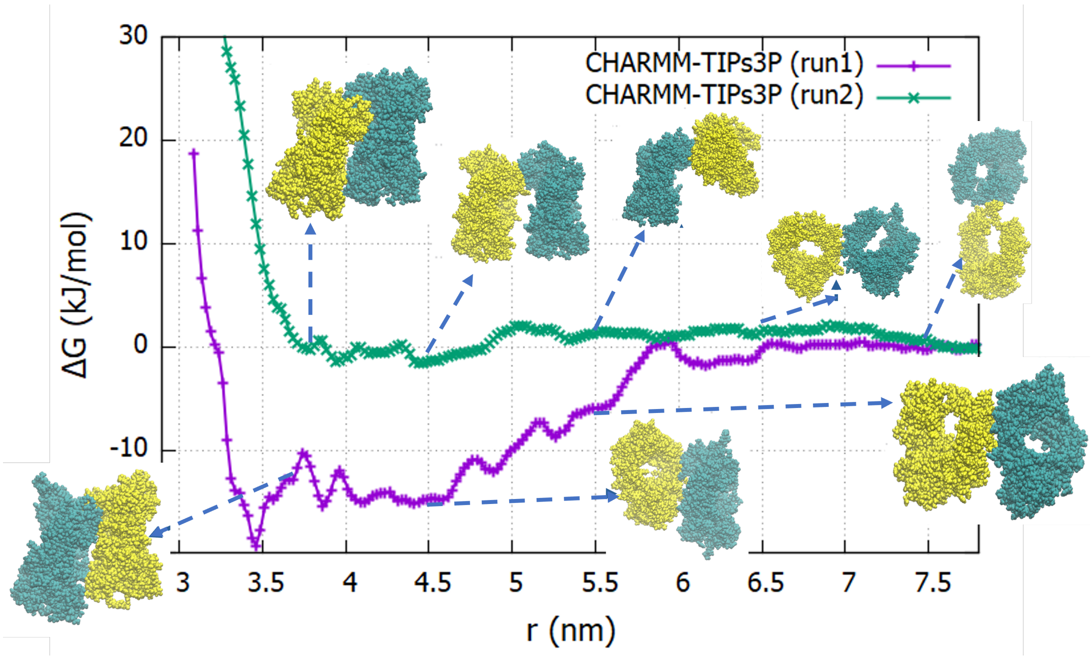

Figure S12.: **Final Fc-Fc conformations for different umbrella windows for the charmm ff system with the tips3p water model. Two different PMF profiles were calculated starting from two different inter-protein conformations.**

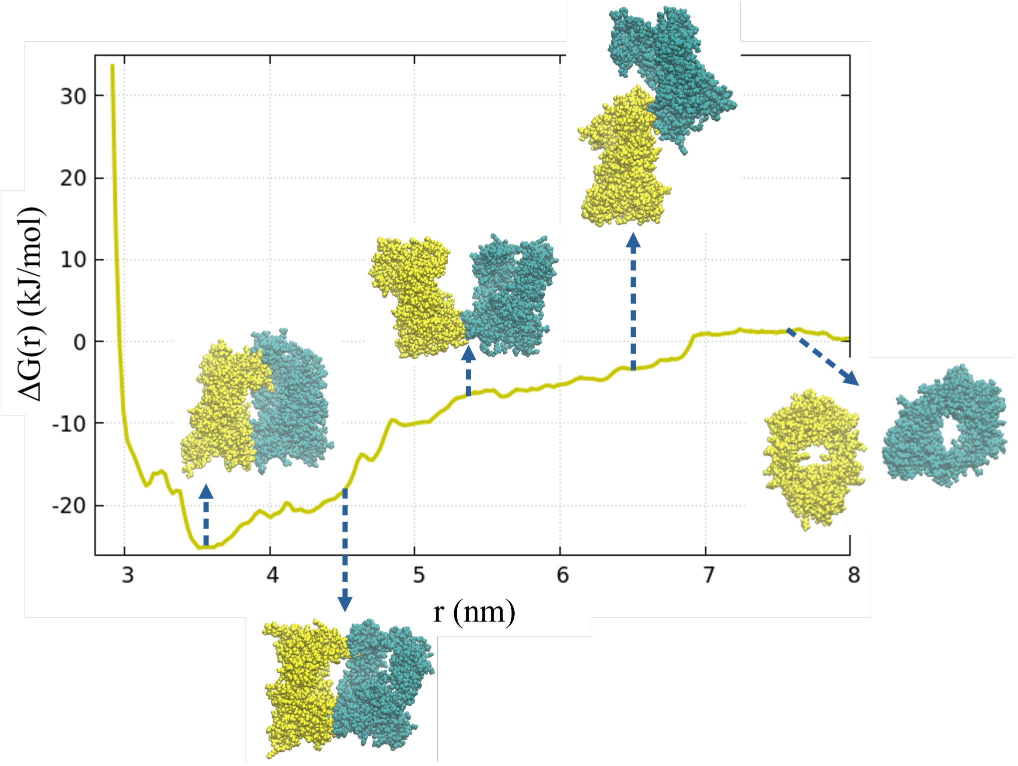

Figure S13.: **Final Fc-Fc conformations for different umbrella windows for the gromos ff system with the spc water model.**

10. Convergence of PMF data

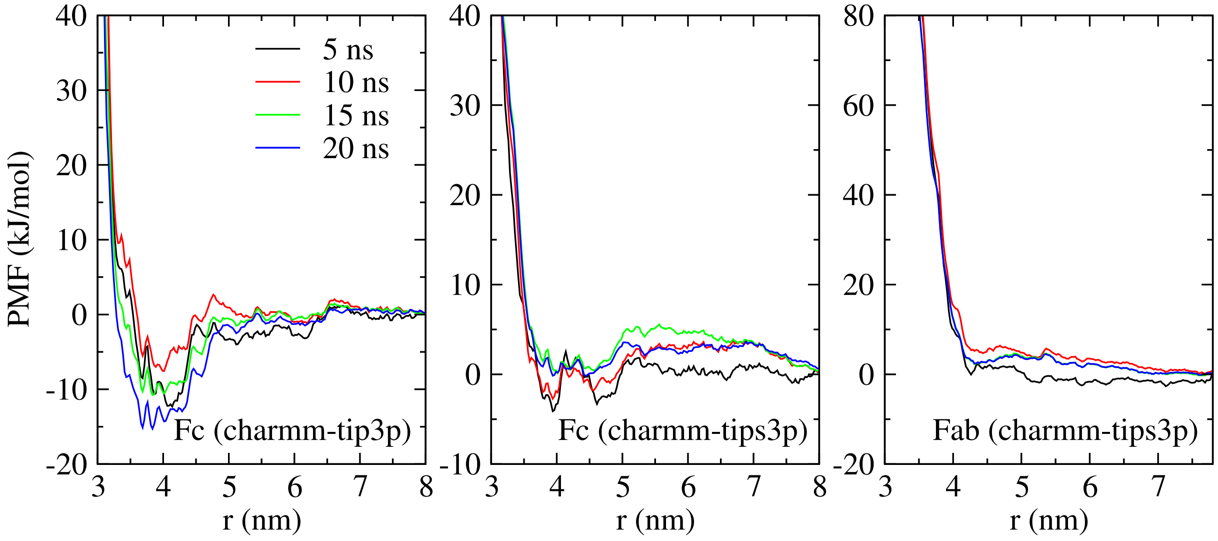

Figure S14.: **The PMF profiles obtained form 5 ns, 10 ns, 15 ns and 20 ns of production run in each umbrella window. The small and non-monotonous variation in the PMF profiles as a function of the simulation time shows that the simulation time of 20 ns in each window is sufficient to obtain converged profiles.**
